# Polypropylene Color Masterbatches Containing Layered Double Hydroxide Modified with Quinacridone and Phthalocyanine Pigments—Rheological, Thermal and Application Properties

**DOI:** 10.3390/ma16186243

**Published:** 2023-09-16

**Authors:** Magdalena Kozłowska, Magdalena Lipińska, Michał Okraska, Joanna Pietrasik

**Affiliations:** Institute of Polymer and Dye Technology, Lodz University of Technology, 90-924 Łódź, Poland; magdalena.stefaniak@dokt.p.lodz.pl (M.K.); michal.okraska@p.lodz.pl (M.O.); joanna.pietrasik@p.lodz.pl (J.P.)

**Keywords:** polypropylene masterbatches, layered double hydroxide, quinacridone pigments, phthalocyanine pigments, viscoelastic properties

## Abstract

Polypropylene color masterbatches containing modified layered double hydroxides, LDHs, were created. The simple, industry-acceptable method of LDH surface modification with quinacridone and phthalocyanine pigments using the pulverization method in ball mills was applied. It was reported that the modification parameters such as time and rotational speed affected the tendency to create the aggregates for modified fillers. TGA analysis of the modified LDH showed that modification with phthalocyanine pigment shifted the temperature at which 5%, T_5%_, and 10% of mass loss, T_10%_, occurred compared with that for unmodified LDH. The viscoelastic properties of prepared masterbatches were investigated. The incorporation of the modified fillers instead of neat pigments led to an increase in the loss shear modulus, G″, indicating a stronger influence on the dissipation of energy by the melted masterbatch. The similar values of tan, δ, were determined for melted masterbatches containing phthalocyanine pigment and green modified LDH filler. The incorporation of both LDHs modified by phthalocyanine and quinacridone pigment fillers slightly increased the zero-shear viscosity, η_0_, compared with that of the masterbatches based on the neat pigments. The Cole–Cole plots and the analysis of the Maxwell and continuous relaxation models showed that modified colored LDH fillers facilitated the relaxation of the melted masterbatch, and shorter relaxation times were observed. The phthalocyanine-modified LDH filler improved the thermal stability of the masterbatches. Additionally, the impact of pigments and modified, colored LDH on the crystallization of polypropylene was investigated.

## 1. Introduction

Polypropylene is one of the most important commodity plastics used in variety of applications, among them fibers [1], composite materials [2,3], and food packaging products [4]. Commonly, additives such as stabilizers and plasticizers [4], fillers [5,6], flame retardants [1] or dyes and pigments [7,8] are incorporated into polypropylene to adjust the mechanical and processing properties and to improve appearance.

Linear trans-quinacridone and phthalocyanine organic pigments have received attention for their mass coloration of the polypropylene fiber [1,9].

Quinacridone, 5,12-dihydroquinolino(2,3-b)acridine-7-14-dione is one of the most important red–violet-shade pigments [10], and due to its photovoltaic activity, efficient emission, effective carrier mobility, it is used in organic thin-film transistors [11]. Quinacridone particles form strong a particle–particle network via intermolecular N-H·····O hydrogen bonding and π–π stacking [12] that results in the insolubility of the quinacridone pigment in common organic solvents and strong difficulty in controlling particle size as well as the aggregation of quinacridone particles after synthesis.

Phthalocyanines are highly conjugated, aromatic, planar macrocyclic organic compounds with eighteen delocalized π-electrons [13,14]. Phthalocyanines exist as both free-metal and metal complexes, and they have been used in applications in catalysis, sensors, light-harvesting dyes in solar cells (organic photovoltaics), and absorbers in non-linear optic and textile dyes [13,14,15]. Phthalocyanine dissolved in s suitable solvent and complexed with heavy metal salts forms organic complexes with bright colors; thus, the commonly used blue and green organic pigments are phthalocyanine pigments [16,17]. Organic phthalocyanine pigments face the same problems as do quinacridone pigments in the process of practical application, including poor dispersion, uneven particle size and easy agglomeration properties leading to poor color quality of the final product.

Polyolefin products, including polypropylene, to be attractive for clients, need to have a desired color. Coloring substances are usually introduced into polymer melts, during the phase of obtaining a granulate (mixing extrusion), before the forming process (injection and extrusion) [18]. Most often, color concentrates, otherwise known as masterbatches, are used for dyeing polymers [19]. White color masterbatches based on titanium dioxide pigments are often used to produce color injection-molded products such as spoons, forks, knives [19,20]. The masterbatches reinforced with nanoclays were designed to produce food trays with barrier properties [21]. Various organic pigments, among them quinacridone pigments, ultramarine blue, diketopyrrolopyrole, are used to prepare polypropylene masterbatches by commercial companies [22]. A color masterbatch is a highly concentrated pigment or a mixture of pigments/dyes enclosed in the form of granules. They may additionally contain additional components, such as antioxidants, antistatic agents, plasticizers, etc. The production process is multi-stage. It starts (in the plasticizing system of the extruder) with the dosing of the ingredients, their melting and mixing with the provision of thermal energy, homogenization and the subsequent cooling of the material ribbon coming out of the head. The last stage is cutting the web into homogeneous granules using a granulator [18].

**Scheme 1:**
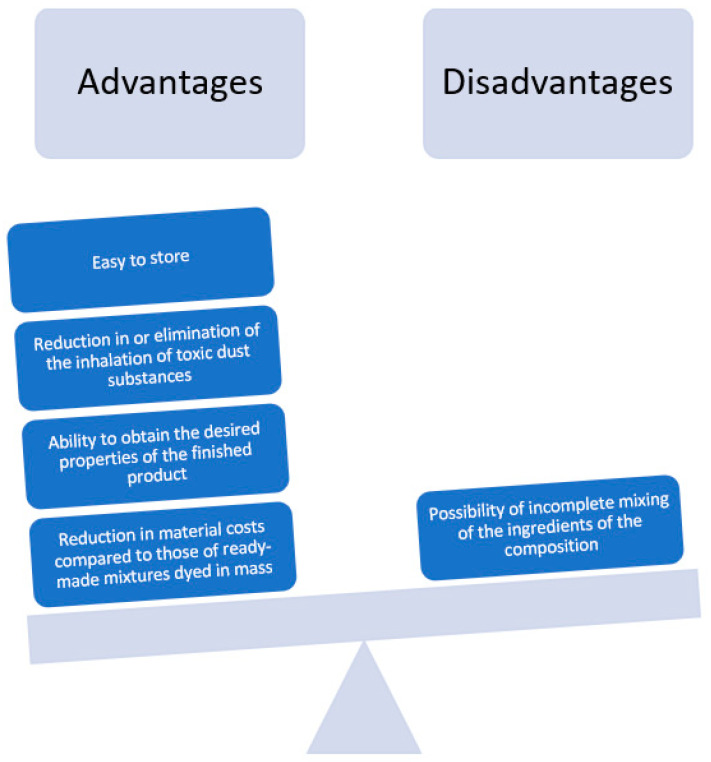
Masterbatch or raw pigment?

The obtained color concentrate offers plastic processors a number of advantages resulting from their use (Scheme 1). First is a reduction in material costs compared to those of ready-made mixtures dyed in mass. Another advantage is the ability to obtain the desired properties of the finished product. An important aspect is a reduction in or the complete elimination of the inhalation of toxic dust, which occurs when powders are used for dyeing.

The biggest disadvantage of using masterbatches is the possibility of the incomplete mixing of the ingredients of the composition. This process shows some non-uniformity in homogenization. The random nature of mixing polymer components to a certain extent, with temporary or permanent effects of the segregation of mixed components, causes significant disturbances in the uniformity of mixed components, and consequently an uneven coloring of the produced product [18]. Therefore, the prevention of pigment aggregation is crucial. The surface free energy and polarity of organic pigments differ from those of polypropylene, which can generate interfacial tension in polar/non-polar systems [23]. To prevent the aggregation of dyes, various strategies were applied, such as the addition of dispersants [24], the grafting of polymers on pigments [25], and the absorption of the pigment on polysaccharide materials via the presence of hydrogen bonding [26]. One of the promising methods is the modification of layered double hydroxides with organic dyes [27].

Layered double hydroxides, LDHs, are composed of positively charged brucite-like layers containing anion and water molecules in the interlayer gallery, with the general formula [M^2+^_1−x_M^3+^x(OH^−^)_2_]^x+^[(A^n−^)_x/n_·mH_2_O]^x−^, where M^2+^ and M^3+^ are cations that occupy octahedral positions in hydroxide layers, A^n−^ [28]. Modified LDHs are able to improve the flame retardancy, thermal stability and combustion behavior of polyolefine material [29]. The Zn–Al LDH was used as inorganic host for the encapsulation of negatively charged anionic dyes; the hybrid organic–inorganic pigment was synthetized using the coprecipitation method [30]. Maragoni R. et al. [31] used layered hydroxide salts intercalated and adsorbed with anionic blue and orange dyes as coloring substances for poly(vinyl alcohol), PVA. Various blue dye molecules were incorporated via the co-precipitation method within the galleries of layered double hydroxide, and blue colored hybrid organic–inorganic pigments were applied as coloring additives to polystyrene PS [27]. The new color-tunable hybrid dark-red to violet pigments based on layered double hydroxides and 1,2-dihydroxyantraquinone dye were obtained via the modification of a filler surface in a water/alcohol dye solution [32]. Coiai S. et al. [33] prepared co-intercalated fluorescent LDH particles via both anion exchange and calcination–rehydration methods using, as modifying substances, fluorescein and alkyl sulfate anions; modification was an effective strategy for preventing the aggregation of the dye. Polypropylene nanocomposites with better tensile strength and enhanced durability under accelerated aging were prepared via the incorporation of layered double hydroxide modified with a UV-absorbing azo-dye, 3(4-anilinophenylazo) benzenesulfonic acid [34].

It can be concluded that it is possible to use layered double hydroxide as a carrier for coloring substances and to produce colored polymer nanocomposites. Thus, in our work colored LDH fillers modified with quinacridone and phthalocyanine pigments were used as the additive to the masterbatch. The industry-acceptable method of modification via the pulverization and application of ball mills was applied to obtain modified fillers. The rheological properties of polypropylene masterbatches containing modified colored LDH fillers were investigated. The impact of chosen color systems on the relaxation behavior of melted polypropylene was reported. It was expected that via the incorporation of the synergistic system LDH/pigment, the thermal properties of the masterbatch would be improved. Additionally, the effect of pigments and modified colored LDH on the crystallization of polypropylene was predicted.

## 2. Materials and Methods

### 2.1. Materials

The layered double hydroxide, LDH, hydrotalcite Pural MG70, product no. 595070, produced by Sasol Germany GMbH (Hamburg, Germany), further in the text denoted as HT, was used to prepare a modified hybrid colored filler. The properties of used hydrotalcite were as follows: a MgO:Al_2_O_3_ ratio of 70:30, and a surface area mg^2^/g, measured using BET methods after calcination for 3 h at 550 °C, of 196 m^2^/g.

The XRD patterns of used hydrotalcite are illustrated in Figure S1 (Supporting Information). The observed peaks were similar to those of the crystallographic pattern described in the literature for Mg–Al layered double hydroxides [35,36]. The existence of an ordered layered structure of the layered double hydroxide was confirmed via the presence of the main sharp and symmetrical peak at 2θ = 11.40° (determined via the 003 reflection in XRD analysis). The interlayer spacing calculated based on the main peak according to Bragg equation [37] was 0.78 nm and did not changed after modification. Similar interlayer spacing was determined by other authors [35] for the unmodified layered double hydroxide containing carbonate anions in the interlayer space. The small characteristic peak was found at 2θ = 5.6°, indicating expanded interlayer spacing (d = 1.58 nm) due to presence of water in the interlayer space [36].

**Figure 1:**
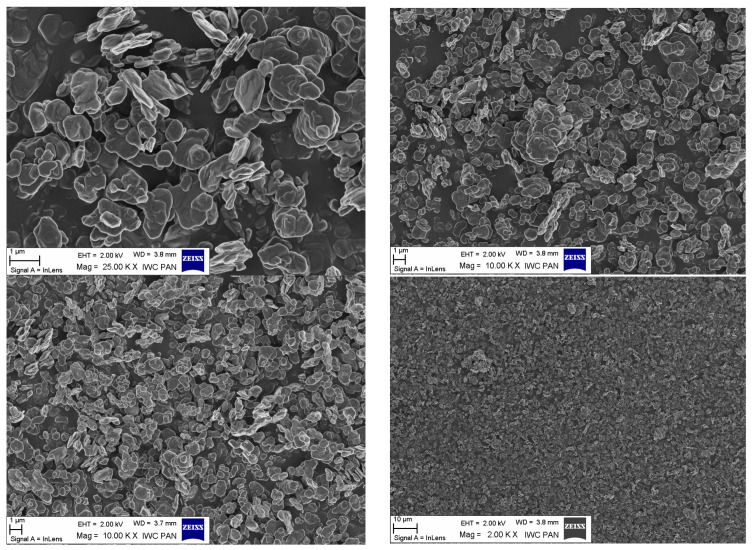
The SEM pictures of the layered double hydroxide, hydrotalcite, HT, used to prepare the hybrid colored filler (SEM microscope, LEO 1530 Gemini, producent Zeiss/Leo, Oberkochen, Germany).

Figure 1 shows the morphology of the HT particles used to prepare the hybrid colored filler.

**Figure 2:**
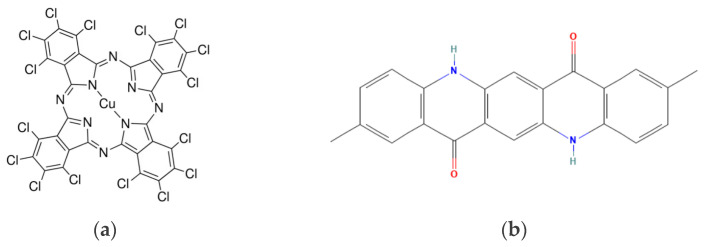
The chemical structures of used pigments: (**a**) pigment Green 7, phthalocyanine green, PG7; (**b**) pigment Red 122, 2,9-dimethylquinacridone, PR122.

Two various pigments were used to prepare the modified hybrid colored HT pigments: (1) pigment Green 7, phthalocyanine green C_32_C_16_CuN_8_, no. CAS: 1328-53-6, producent Sigma-Aldrich (Poznań, Poland), further denoted as PG7; (2) pigment Red 122, 2,9-dimethylquinacridone,((2,9-dimethyl-5,12-dihydroquinolino [2,3-b]acridine-7,14-dione), C_22_H_16_N_2_O_2_, no. CAS 980-26-7, producent Sigma-Aldrich (Poznań, Poland), further denoted as PR122. The chemical structures of the used pigments are shown in Figure 2.

### 2.2. Preparation of Modified Colored Filler

**Scheme 2:**
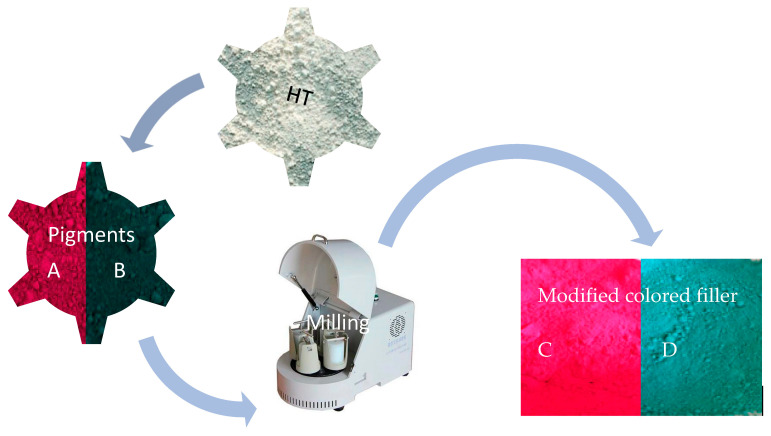
Preparation of modified colored filler. A—pigment Red 122, 2,9-dimethylquinacridone,((2,9-dimethyl-5,12-dihydroquinolino[2,3-b]acridine-7,14-dione), PR122; B—pure pigment Green 7, phthalocyanine green, PG7; C—modified colored filler HT-Red 50 rpm; D—modified colored filler HT—Green 50 rpm.

The modification was carried out by using the PM 200 planetary-ball mill (Pulverisette 5, Fritsch-GmBH, Idar-Oberstein, Germany) according to the Scheme 2. First, placing 10 g of pigment and 100 g of filler was placed in a steel vessel with grinding balls. A rotational speed of 50 rpm for 15 min was set, or 60 rpm for 10 min in order to find more favorable mixing parameters in terms of energy. In this case, the following sample designations were adopted: HT-Green 50 rpm, 60 rpm and HT-Red 50 rpm, 60 rpm for hybrid colored pigments modified, respectively by phthalocyanine green and 2,9-dimethylquinacridone using procedure described above.

The ball milling process is a green technology regarded as a “top-down” approach in the production of fine particles. The ball milling process can be used to increase the particle surface area and further to optimize the dispersion state of particles in biocomposites [38]. The mechanical energy created during milling allows the physical breakdown of coarse particles into finer ones; thus, it is used to produce fine drug particles [39]. Ball milling technology can be used for the preparation and chemical functionalization of polymers, e.g., nanocellulose derivatives [40]. Similarly, various milling techniques are applied for the simultaneous attainment of particle dispersion and the surface modification of solids such as metal oxides [41] or layered fillers [42]. The simplicity and low cost of filler modification using ball mills compared with other methods of filler surface modification, e.g., the solvent method, are great advantages for the industry. Among other surface modification methods, it allows an avoidance of the use of organic solvents and thus is regarded as being an environmentally friendly technique [40]. Here, it is also important that different types of ball milling equipment are available at low prices. Thus, we selected this method as an easy-to-use, fast, profitable and ecological method to modify the surface of the layered double hydroxide using selected pigments.

### 2.3. Preparation of Masterbatches

The carrier of the coloring substance was polypropylene, PP. As a polymeric carrier of the masterbatch, the polypropylene Sabic PP520P (Sabic Europe, Geelen, The Netherlands), particularly designed for the extrusion of cast films, and typically used for food, hygiene and textile packaging and lamination films, was used. The parameters of the PP were a melt flow rate, MFR, of 10.5 g·min^−1^ at 230 °C and 2.16 kg (ASTM D 1238 [43]), and a density of 905 kg·m^−3^ (ASTM D792 [44]).

The modified colored pigments HT-Green and HT-Red were added to the masterbatch as a replacement for the neat pigment in the masterbatch composition. The amount of incorporated modified pigments and neat pigments was 0.25 g for 100 g of matrix polypropylene for every masterbatch produced. The prepared samples were further denoted as follows: PP—neat polypropylene sample; PP/HT—polypropylene sample containing unmodified layered double hydroxide; PP/PG7—masterbatch containing neat pigment Green 7, phthalocyanine green; PP/PR122—masterbatch containing neat pigment Red 122, 2,9-dimethylquinacridone; PP/HT-Green—masterbatch containing layered double hydroxide modified with phthalocyanine green; PP/HT-Red—masterbatch containing layered double hydroxide modified with 2,9-dimethylquinacridone.

**Scheme 3:**
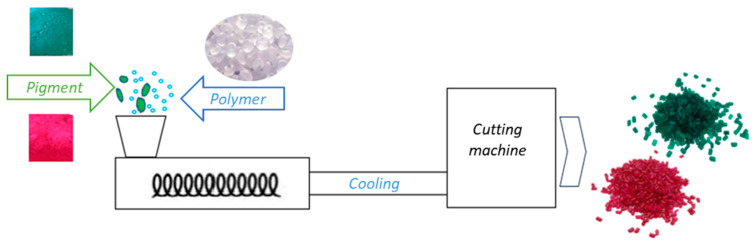
Production of polypropylene color concentrate containing modified HT. C—modified colored filler HT-Red 50 rpm; D—modified colored filler HT—Green 50 rpm; E—masterbatch PP/HT-Red 50 rpm.; F—masterbatch PP/HT-Green 50 rpm.

The production of color concentrates was achieved using a single-screw extruder at 220 °C. The method of their preparation is presented in the diagram below (Scheme 3).

Additionally, the test samples for viscoelastic studies in the form of plates with a thickness of 2 mm were made by using a single-screw extruder and an injection molding machine.

### 2.4. The Characterization of Modified Colored LDH Fillers

To estimate the tendency toward aggregation after modification, aggregate size analysis of the prepared modified fillers was performed by using the dynamic light scattering, DLS, technique. The size of the unmodified layered double hydroxide HT, neat pigments PG7 and PR122 and modified colored fillers HT-Green and HT-Red was determined using Zetasizer Nano Serie S90 (Malvern Panalytical Ltd., Malvern, UK). The size of the particles was measured for the water dispersions, and the concentration of the dispersion was 5 g of modified filler per 100 mL of dispersing medium. To estimate the tendency of modified colored fillers in a non-polar medium, the size of the agglomerates was additionally measured in paraffin oil (model of polypropylene); paraffin oil produced by PHU Olmax S.J., Lodz, Poland was used. Before the measurements the dispersions were stabilized via ultrasonic treatment for 10 min (Ultrasonic bath, Bandelin Sonorex DT 255, Bandelin GmbH, Berlin, Germany).

The optical microscope images were obtained using the Opta-Tech Lab40 microscope (Opta-Tech, Warsaw, Poland) connected with digital camera Mi6 with 6 megapixel sensor-IMX178, Resolution -3072 × 2048 (Opta-Tech, Warsaw, Poland), and computer program Capture 2.3 (Opta-Tech, Warsaw, Poland). The results of the studies are compiled in the Supporting Information, Figures S2–S4.

The surface energy of modified colored fillers was calculated using the Owens–Wendt–Rabel–Kaelble, work, method [23,45]. The Owens–Wendt–Rabel–Kaelble method is the standard procedure for calculating the surface free energy of a solid. The tested solid is wetted with several measuring liquids, which enables the division of the surface energy into two components: polar and dispersive. The OWRK method enables the optimization and observation of polarization changes between two surfaces. The test was performed at ambient temperature using the K100 MKII tensiometer (KRÜSS GmbH, Hamburg, Germany). Polar (water and chloroform) and non-polar (1,4-dioxane) liquids were used. At least three measurements were taken for each testing liquid. In the addition, the polarity was calculated based on the simple equation polarity = *γ*^p^/*γ*, where *γ*^p^ is the polar component (dynes·cm^−1^) and *γ* is the total free energy (dynes·cm^−1^).

### 2.5. Viscoelastic Properties at Processing Temperature 200 °C

The dynamic viscoelastic properties of PP masterbatch mixtures were studied at 200 °C. A similar temperature for the rheology tests was used by other authors [5] for polypropylene composites containing layered double hydroxides. The oscillation rheometer Ares G2 (TA Instruments, New Castle, DE, USA) equipped with a plate–plate geometry (diameter: 25 mm) was used during the tests.

To perform oscillation testing in the plate–plate geometry, the sample was loaded between plates and it oscillated back and forth at a given stress or strain amplitude and frequency. The applied motion can be represented as a sinusoidal wave with the stress or strain amplitude. The ratio of the applied stress (or strain) to the measured strain (or stress) is a quantitative measure of material stiffness, and it gives the complex modulus, G* (Equation (1)) [46].

(1) $$G^{*}=\frac{\sigma_{max}}{\gamma_{max}}$$

For an elastic material (stress is proportional to strain), the maximum stress occurs at the maximum strain and both stress and strain are said to be in-phase. For viscous materials, stress and strain are out-of-phase by 90° or π/2 radians. For viscoelastic materials, the phase difference (the phase angle δ) between stress and strain is between two extremes. This phase difference allows the viscous (loss modulus, G″) and elastic component (storage modulus, G′) ratio to the total material stiffness (G*) to be determined in accordance with the equation (Equation (2)):

(2) $$G^{*}=\sqrt{({G^{\prime }}^{2}+{G^{\prime \prime }}^{2}});\;G^{\prime }=G^{*}cos\delta ;\;G^{\prime \prime }=G^{*}sin\delta$$

The relationship can also be presented in the form of Equation (3):

(3) $$G^{*}=G^{\prime }+iG^{\prime \prime }$$

where i is an imaginary number equal to $\sqrt{1}$.

Complex viscosity, η*, is a measure of the total resistance to flow as a function of angular frequency (ω) and it is calculated in accordance with Equation (4):

(4) $$\eta^{*}=\frac{G^{*}}{\omega }$$

It can be divided into two component parts, that include dynamic viscosity, η′, and the out-of-phase component of complex viscosity, η″. Both represent the real and the imaginary parts of η*, respectively, in the following form (Equation (5)):

(5) $$\eta^{*}=\eta^{\prime }+i\eta^{\prime \prime }$$

The amplitude sweep tests at 200 °C, at a constant value of angular frequency, 10 rads^−1^, were performed; based on the tests the averages values of the storage shear modulus, G′, loss shear modulus, G″, and loss factor tan, δ, for the linear viscoelastic region were calculated. The frequency sweep tests at 200 °C, at a constant value of the oscillation amplitude, 0.5%, for a varied frequency range, 0.1–628 rad·s^−1^, were performed.

The analysis of the relaxation of the melted masterbatches containing neat hydrotalcite, HT, particles and pigment-modified HT was performed based on the frequency sweep test using various relaxation models.

The Maxwell model [47] was applied. The linear viscoelastic data were recalculated by using Equations (6) and (7). The discrete relaxation spectrum was obtained, and the relaxation times, λ_i_, and values of the relaxation modulus, G_i_, were calculated.

(6) $$G^{\prime }\left(\omega \right)=\sum_{i=1}^{N}G_{i}\frac{(\omega \lambda_{i}{)}^{2}}{1+(\omega \lambda_{i}{)}^{2}}$$

(7) $$G^{\prime \prime }\left(\omega \right)=\sum_{i=1}^{N}G_{i}\frac{\left(\omega \lambda_{i}\right)}{1+(\omega \lambda_{i}{)}^{2}}$$

The six Maxwell elements were sufficient for the recalculation of the values of the storage, G′, and loss shear modulus, G″, for the frequency sweep experimental data. The level of correlation was R^2^ = 0.999.

The following model equations, Equations (8) and (9) [48,49,50], with n terms were used to extract the continuous relaxation spectrum by fitting the oscillation data.

(8) $$G^{\prime }\left(\omega \right)=\int_{-\omega }^{+\omega }H(\ln\tau )\frac{\omega^{2}\tau^{2}}{1+\omega^{2}\tau^{2}}d\ln\tau$$

(9) $$G^{\prime \prime }\left(\omega \right)=\int_{-\omega }^{+\omega }H(\ln\tau )\frac{\omega \tau }{1+\omega^{2}\tau^{2}}d\ln\tau$$

The spectrum, H(lnτ), was discretized in the order of 100 steps. The spectrum represents all the pairs of fitted {Hi,τi} parameters.

The numerical fitting of the storage shear modulus, G′, and loss shear modulus, G″, to the relaxation models was conducted using the TRIOS^®^ Software (TRIOS v3 1.5.3696) provided by TA Instruments (New Castle, DE, USA).

Additionally, the zero-shear viscosity, η_0_, and characteristic mean relaxation times, τ_m_, were calculated from viscosity Cole–Cole plots (plots of η″ vs. η′, where η′ is dynamic viscosity and η″ is the out-of-phase component of complex viscosity, η*) as proposed in the literature [51,52,53,54].

### 2.6. The DSC and TGA Analysis

DSC analysis was performed using a DSC1 apparatus (Mettler Toledo, Ithaca, NY, USA). All tests were performed in a nitrogen atmosphere. The samples were subjected to three heating/cooling/heating steps from −150 °C to 200 °C with a heating rate of 10 °C·min^−1^. The first heating step was performed to eliminate the thermal history of the sample. The degree of crystallinity, χ_c_, was calculated from both the first and second cycle of heating in accordance with Equation (6) [55,56,57].

(10) $$\chi_{c}=\frac{\Delta H_{m}}{{\Delta H}_{m}^{0}}\cdot \frac{100}{w}$$

where ΔH_m_ is the experimental melting enthalpy, ${\Delta H}_{m}^{0}$ is the the enthalpy of melting of 100% crystalline PP (207.1 Jg^−1^ [55,56,57]), and w is the weight fraction of polypropylene.

TGA analysis was performed using a TGA/DSC1 (Mettler Toledo, USA) analyzer. Samples were heated from 25 °C to 600 °C in argon, and from 600 °C to 900 °C in air, with a heating rate of 10 °C·min^−1^.

### 2.7. CIELab Measurements

In order to determine the influence of the hybrid pigment on the color profile of the masterbatch, the CIELab model was used, where the lightness of color is L with a scale from 0 (black) to 100 (white). Two coordinates, a,b, can take both positive and negative values. Positive values of the *a* coordinate determine the share of red, and negative value determine the share of green green. Positive values of the *b* coordinate refer to the share of yellow, and negative values refer to the share of blue. The difference between two colors in the space is calculated on the basis of a mathematical formula (Equation (11)):

(11) $$∆E=\sqrt{{\left(∆L\right)}^{2}+{\left(∆a\right)}^{2}+{\left(∆b\right)}^{2}}$$

The measurements were taken using a Konica Minolta CM-36dG apparatus (Konica Minolta Inc. Japan, Chyoda-Tokio, Japan).

## 3. Results

### 3.1. Surface Energy, the Tendency toward Aggregation and the Thermal Stability of Colored Layered Double Hydroxide Fillers

Layered double hydroxides demonstrate a strong tendency to agglomerate due to electrostatic interactions and the possibility to form hydrogen bonds via hydroxyl groups present on the filler surface. The particle size and the distribution of the filler aggregates in the polymer matrix can be important factors influencing the rheological behavior of filled polymer materials. Uneven dispersion and the occurrence of large agglomerates can be disadvantages from the processing point of view. They can generate problems during the further mixing of a masterbatch with the polymer as well as affect the color stability of the masterbatch. The modification of the surface can both reduce the tendency toward agglomeration or enhance the formation of aggregates. Therefore, the aggregate size in water and paraffin oil for modified colored fillers was studied to estimate the influence of modification on the tendency to agglomerate in polar and non-polar mediums.

The modification of layered double hydroxide with pigments influenced the size of the formed aggregates in the water medium (Table 1). Hydrotalcite modification with both PG7 and PR122 pigments resulted in an increase in the range of the aggregate size in the polar medium. A much higher tendency of HT-Green toward aggregation was noticed than that of the composition of HT-Red.

After the modification of the layered double hydroxide surface with PG7 pigment, the range of the formed aggregates in the non-polar paraffin oil medium increased, but still the formed aggregates were not bigger than 3000 nm (Table 2). Oppositely, for HT-Red fillers modified with PR122 pigment a lower tendency toward agglomeration was indicated. It should be noted that PG7 is a polar compound and due to presence of chloride atoms in the structure its surface free energy strongly differs from that of the non-polar medium resulting in worse stability of the dispersion. The DLS plots of aggregate sizes as a function of the percentage by volume are shown in Figures S5 and S6 in the Supporting Information.

The optical microscope images were taken for pure hydrotalcite, HT, and neat pigments before milling (Figure S2, Supporting Information) as well as for HT–pigment powders after milling at various milling speeds (Figures S3 and S4, Supporting Information). The optical microscopy studies confirmed that the hydrotalcite before milling formed grains, with diameters larger than 80 μm. Both pigments formed smaller aggregated grains, but still the agglomerates with diameters larger than 80 μm were present. The optical microscopy analysis confirmed that after the modification, phthalocyanine green PG7 pigment was present mostly on the surface of the hydrotalcite grains, and did not form separated grains. Milling at a 50 rpm speed for 15 min reduced the diameter of the largest aggregates. Oppositely, for HT-Green 60 rpm, larger aggregates were observed. This is in agreement with the findings of DLS studies. For HT-Green 60 rpm, larger aggregates were also determined to be present in water as well as in the non-polar paraffine oil medium compared with those with HT-Green 50 rpm. Similarly, for the modified HT-Red fillers, pigment was mostly present on the surface of HT grains (Figure S4, Supporting Information). A reduction in the size of HT-Red grains was observed independently of the applied speed of milling.

The surface free energies of tested samples were In the range of 10.7–27.8 dynes·cm^−1^ (Table 3). The calculated polarity index was from 0.05 up to 0.44. The polar component for PG7 and PR122 was due to their chemical structure. All tested modified hydrotalcite fillers showed a more non-polar character. It is worth noting the decrease in the polar composition of HT-Green 50 rpm and HT-Red 50 rpm with respect to unmodified HT and pure pigments PG7 and PR122, which is beneficial from the point of view of mixing them with non-polar polypropylene, PP.

**Figure 3:**
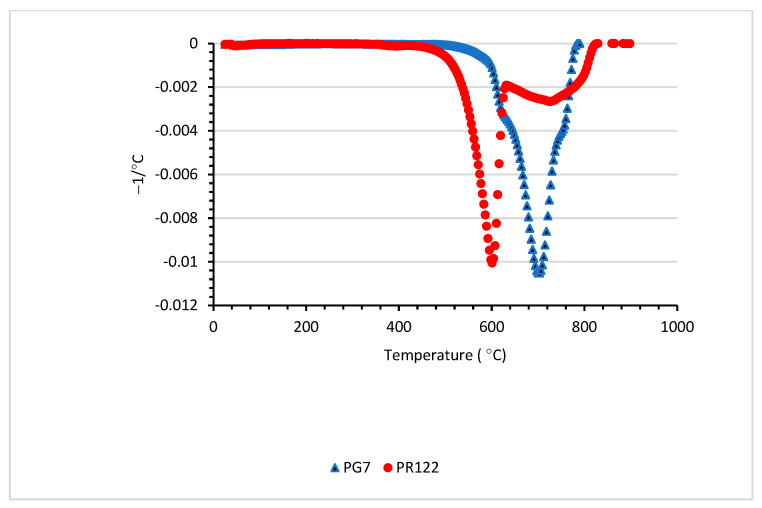
DTGA plots of neat PG7 and PR122 pigments.

**Figure 4:**
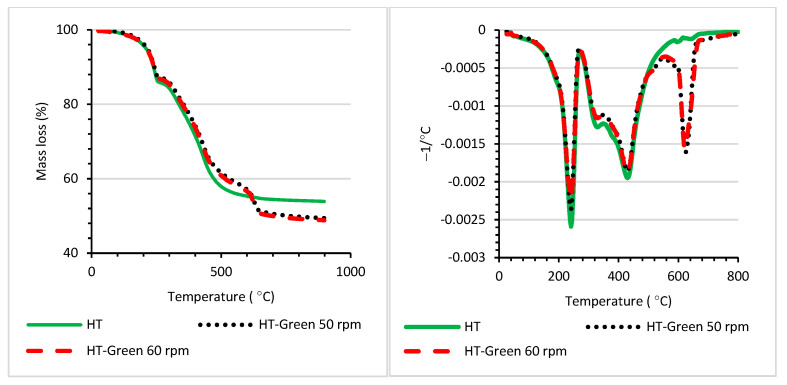
TGA and DTGA plots of HT-Green fillers.

The thermal stability of the obtained colored fillers was analyzed using the TGA method (Figure 3 and Figure 4). The DTGA plots for neat PG7 and PR122 confirmed the high thermal stability of both pigments. The degradation and weight loss for the PG7 pigment was reported in the temperature range of 520–780 °C and for that for the PR122 pigment was reported in the temperature range of 450–820 °C (Figure 3). A two-step mechanism of mass loss was observed for the PR122 pigment, identified as two peaks in the DTGA plots, with the maximum occurring at 601 °C and 733 °C. A single degradation peak was observed for the PG7 pigment with a maximum at 697 °C.

**Figure 5:**
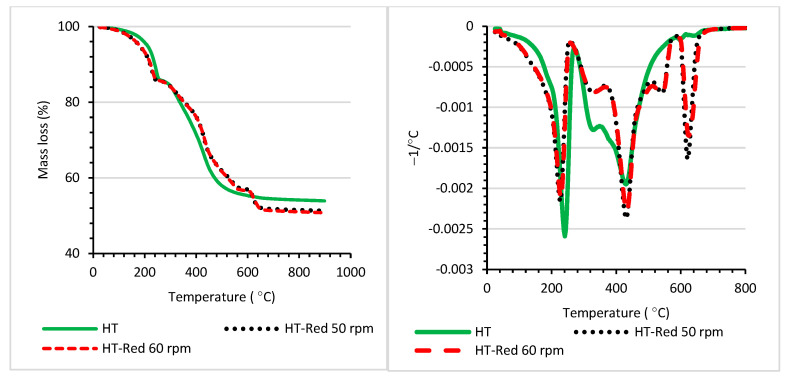
TGA and DTGA plots of HT-Red fillers.

The temperatures at 5, 10, 20% of weight loss, and the temperatures at the maximum of the DTA peaks for the studied pigments and colored fillers are compiled in Table 4. The thermogravimetric analyses (TGA) of unmodified hydrotalcite, HT, and modified, colored fillers are presented in Figure 4 and Figure 5.

Two loss regions at the temperature range of 30–270 °C and of 300–600 °C characteristic of hydrotalcite can be seen for the neat and modified fillers. The first mass loss region is attributed to physically and chemically bonded water and the second to the interlayer anions and interlayer water associated with the anions. Similar behavior, and a two-step mechanism of mass loss was observed by other authors for various layered double hydroxides [58]. An additional loss mass region at a temperature higher than 600 °C was observed for HT-Green and HT-Red fillers, and it was attributed to the thermal decomposition of adsorbed pigments on the filler surface. The layered double hydroxides HT-Green 50 rpm and HT-Green 60 rpm modified with PG7 pigment started to lose water below 95 °C and the observed mass loss was lower than that for the unmodified layered double hydroxide. Further, also, the mass loss observed for the second region, attributed to the removal of the interlayer water, was lower.

The layered double hydroxides HT-Red 50 rpm and HT-Red 60 rpm modified with PR122 pigment started to lose water at around 100 °C, but the dehydration and the removal of physically associated water occurred faster and the mass loss in first region was stronger than that for unmodified layered double hydroxide. The modification of the layered double hydroxide with pigment PR122 strongly influenced the thermal behavior of the HT-Red fillers in the second region, and this was attributed to the removal of the interlayer water associated with anions present in the interlayer space. The removal of the interlayer water started at a higher temperature. Probably, the PR122 pigment was not only adsorbed on the outer layer of the filler via interactions with hydroxyl groups present on the layered double hydroxide surface, but was also able to form associations with the interlayer water.

### 3.2. The Viscoelastic Properties and Relaxation Behavior of the Melted Masterbatches at a Processing Temperature of 200 °C

Dynamic rheological tests are a very sensitive method that allow an estimation of changes in the rheological behavior of a melted polymer after the incorporation of additives. The viscoelastic properties of the masterbatches at a processing temperature of 200 °C were investigated; the storage shear modulus, G′, and the loss shear modulus, G″, were determined as functions of angular frequency and are compiled in Figures S7 and S8 in the Supporting Information.

The incorporation into the masterbatch of the additives, layered double hydroxide HT, pigment PG7 or PR122 and modified HT-Green and HT-Red pigments did not strongly influence the viscoelastic properties of melted the polypropylene, PP. The values of the storage shear modulus, G′, and loss modulus, G″, for the PP compositions containing layered double hydroxide HT or the modified pigment based on the HT filler slightly decreased compared with those of neat PP. Various factors should be considered here; first, the process of masterbatch production via the extrusion of the composition could have influenced the changes in the viscosity of the base polymeric material, polypropylene. As we show in Figure S9 (Supporting Information), for samples studied at 220 °C the extrusion and granulation of the pure palettes of polypropylene under similar conditions as those used in masterbatch production slightly decreased the viscosity of the melted material. Second, the additives, especially pigments, could have influenced the processes of degradation but here more in-depth studies are needed to estimate the effect of pigments on the possible thermo-mechanical or thermo-oxidative degradation of polypropylene. As we analyzed further, the changes in viscosity occurred to a higher extent for the masterbatches containing pure pigments. Another factor influencing the values of the storage modulus, G′, might have been the enhanced mobility (relaxation) of confined polymeric chains at the interface of the PP–LDH layer. This effect was reported by other authors [5] and attributed to the reduced values of the storage shear modulus, G’, for polypropylene layered double hydroxide composites under low layered double hydroxide loading.

From the industrial point of view, what is more important is that the color additives or changes in the composition do not significantly affect the rheological behavior in the melt state of the produced masterbatch.

The average values of the viscoelastic parameters, storage modulus, G′ (Pa), loss modulus, G″ (Pa), and loss factor, tan δ (−), measured at 200 °C at 10 rad·s are compiled in Table 5.

Considering the viscoelastic properties, it can be concluded that pigment-modified HT could have been applied as an additive to the masterbatch instead of neat pigments PG7 and PR122. The storage shear modulus, G′, of the masterbatches containing HT-Green and HT-Red fillers were slightly higher compare with those of the masterbatch based on the neat pigments. A stronger impact of the HT-Green and HT-Red prepared by using a 50 rpm speed of milling on the values of the loss shear modulus, G″, was observed; this was because the dissipation of energy could have been affected by the presence of solid particles. Further, the state of the dispersion of the filler in the melt and its tendency to agglomerate could have been a factor influencing the dissipation of energy by the melted masterbatch. The application of various milling speeds during the preparation of modified HT fillers affected the tendency of the modified HT to form aggregates, as was analyzed in the previous paragraph. It affected the viscoelastic behavior of the melted composition, leading to the higher values of the storage shear modulus, G′, and loss modulus, G″, observed for the compositions based on HT-Green 50 rpm and HT-Red 50 rpm.

The similar values of tan, δ (Table 5), were determined for compositions containing PG7 pigment and HT-Green. Differently, higher values of tan, δ, were reported for the masterbatch containing pure PR122 pigment compared with those of compositions containing modified HT-Red 50 rpm. The higher mixing speed during the modification of HT could have been the factor leading to the reduced size of the formed aggregates. The viscoelastic properties, storage modulus, G′, and loss modulus, G″, of the masterbatch, containing the modified HT-Red filler prepared using a higher speed of mixing were similar to those of the composition containing pure pigment PR122. This confirmed that the optimization of the process of the modification of the filler during the preparation of colored HT-Red fillers was necessary, leading to a reduction in the tendency of aggregation, and further influenced the viscoelastic behavior of the masterbatch containing the modified HT-Red 60 rpm filler.

**Figure 6:**
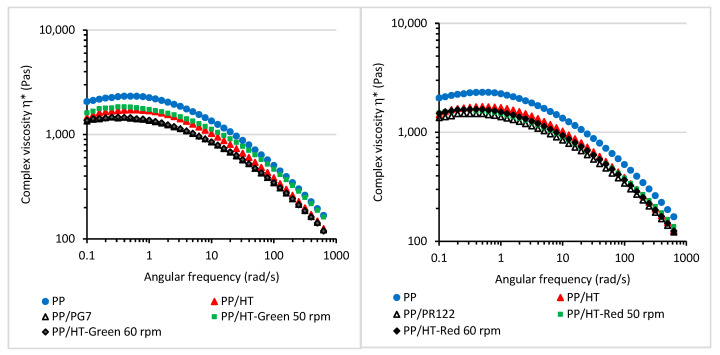
Complex viscosity, η* (Pas), at 200 °C, and at an applied oscillation strain of 0.5%.

The viscosity of the material in the melt state was an important processing parameter. The influence of the modified colored HT on the complex viscosity, η*, dynamic viscosity, η′, and out-of-phase component of complex viscosity, η″, was investigated. The values of complex viscosity, η*, measured as a function of angular frequency at 200 °C are shown in Figure 6.

The plots of complex viscosity, η*, showed a plateau, which is typical for thermoplastic homopolymers at low values of frequency, and shear tinning behavior as the frequency was increased. The values of the complex viscosity, η*, at 200 °C for all compositions containing HT or modified colored HT were lower compared with those of neat PP. The lower viscosity of the studied compositions resulted from the reduction in the viscosity of the polypropylene during masterbatch preparation. The preparation of the masterbatch compositions was conducted in two steps. First, the components of the masterbatch recipe were mixed with PP using an extruder. The obtained material was granulated. Then, the colored pallets were used as an additive to the pure PP thermoplastic material during injection molding. The process of masterbatch preparation could have influenced the viscoelastic properties, leading to the changes in the viscosity of the thermoplastic PP base. During extrusion, the thermo-mechanical and thermo-oxidative aging of the material could have occurred due to the presence of oxygen. The applied shear rate could have resulted in the chain scission of the material and the changes in the dispersity of the material and its molecular weight leading to changes in viscosity. The presence of additives, especially pigments, can promote the thermo-oxidative aging of a material, leading to stronger changes in viscosity during the processing of a masterbatch. The values of complex viscosity, η*, measured for the plateau region (frequency range 0.1–1 rads^−1^) were as follows: PP η* = 2252 ± 68 Pa·s; PP/HT η* = 1657 ± 63 Pa·s; PP/PG7 η* = 1421 ± 32 Pa·s; PP/HT-Green 50 rpm η* = 1778 ± 47 Pa·s; PP/HT-Green 60 rpm η* = 1432 ± 28 Pa·s; PP/PR122 η* = 1449 ± 35 Pa·s; PP/HT-Red 50 rpm η* = 1560 ± 35 Pa·s; PP/HT-Red 60 rpm η* = 1587 ± 33 Pa·s.

**Figure 7:**
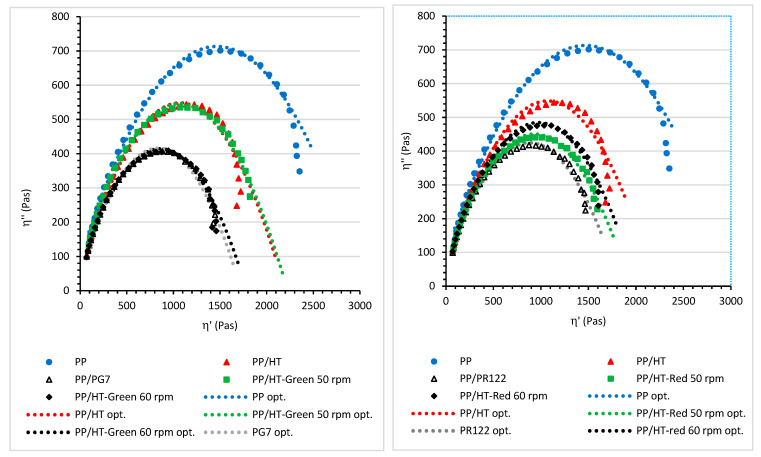
The Cole–Cole plots at 200 °C; η′—dynamic viscosity (Pas), η″—out of phase component of complex viscosity η* (Pas).

The influence of the various additives on the relaxation behavior of the melted polymer and its viscoelastic properties can be estimated based on the viscosity Cole–Cole plots. The viscosity Cole–Cole plots are the plots of out of phase component of complex viscosity η* versus dynamic viscosity η′′. For the homopolymers the Cole–Cole plots usually form the semicircular arcs and the rheological parameters such as the zero-shear viscosity η_0_ and characteristic relaxation time can be derived [50,51,52,53]. The Cole–Cole plots are depicted in Figure 7.

All compositions showed Cole–Cole plots of a semicircular shape, which is typical of homopolymers. The incorporation of layered double hydroxide HT shifted the maximum of the arc compared with that of neat PP. The relaxation of the melted polypropylene was affected by the presence of layered double hydroxide particles. The maximum of the arc was presented at lower values of dynamic viscosity, η′, and at a higher value of angular frequency (a shorter relaxation time). The incorporation of HT particles facilitated the relaxation of melted PP. Further, both pigments, PG7 and PR122, strongly facilitated the relaxation of the masterbatch. The maximum of the arc was shifted to a meaningfully lower value of dynamic viscosity, η′, after the addition of the PG7 pigment. The modification of the layered double hydroxide surface by pigments caused a further facilitation of the relaxation of the masterbatch compared with that of unmodified HT. The selected additives reduced the values of dynamic viscosity, η′. The values of the characteristic relaxation time, τ_m_, and the zero-shear viscosity, η_0_, calculated based on the Cole–Cole plot are compiled in Table 6.

**Figure 8:**
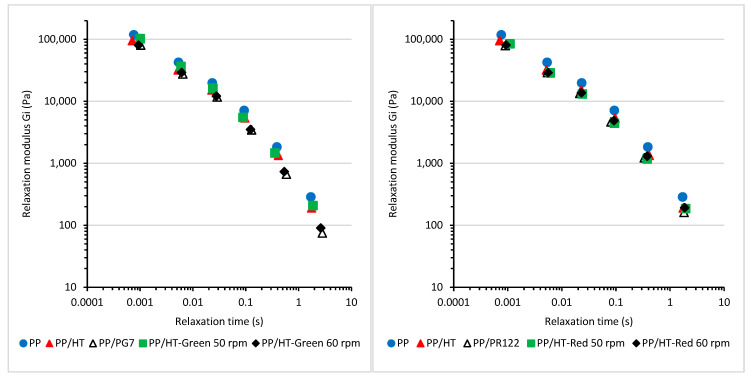
The discrete relaxation spectra calculated using the Maxwell model.

The Maxwell model [47] was applied to calculate the discrete relaxation spectra of the studied masterbatches. The calculated values of the relaxation modulus, G_i_, and relaxation times, λ_I_, are compiled in Tables S1 and S2 (Supporting Information) and depicted in Figure 8.

A more significant influence of the additives on the relaxation spectra was observed for the masterbatches containing the modified HT-Red filler, and lower values of the relaxation modulus, G_i_, and shorter relaxation times, λ_I_, confirmed the facilitated relaxation of the melted PP in the presence of both HT-Red 50 rpm and HT-Red 60 rpm. A similar effect was observed when the modified HT-Green filler prepared using a higher mixing speed (60 rpm) was incorporated into the masterbatch. The incorporation of the unmodified layered double hydroxide slightly increased the values of the relaxation modulus, G_i_. Both PG7 and PR122 pigments slightly facilitated the relaxation of the masterbatch. The effect on the relaxation times was less evident than that for the alternative modified coloring fillers HT-Green and HT-Red.

**Figure 9:**
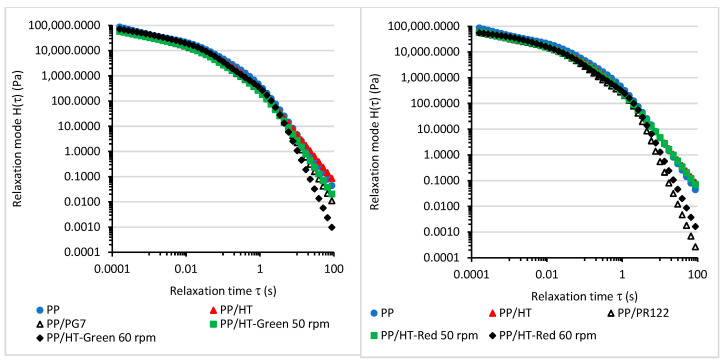
Continuous relaxation spectra for the masterbatches melted at 200 °C.

The influence of the modified colored fillers on the relaxation of the melted masterbatches was confirmed via the calculation, based on the frequency sweep tests, of the continuous relaxation spectra, which are shown in Figure 9.

The incorporation of the both HT-Green and HT-Red instead of the neat pigments PG7 and PR122 facilitated the relaxation of the melted masterbatches, and the reduction in the values of the relaxation modulus, H(τ), occurred faster. This is a processing advantage, and the faster relaxation of the material together with the lower values of the storage shear modulus in a low frequency range were the crucial factors influencing the processing of the material during extrusion, reducing the die-swell of the masterbatch.

### 3.3. Thermal Properties and Crystallization of Masterbatches

**Figure 10:**
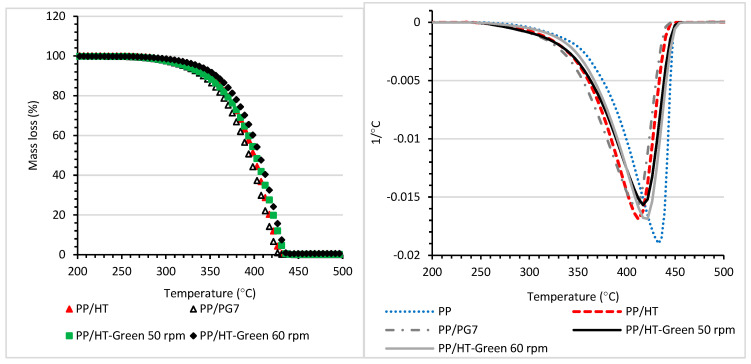
TGA and DTGA plots of masterbatches containing PG7 pigment and modified HT-Green fillers.

**Figure 11:**
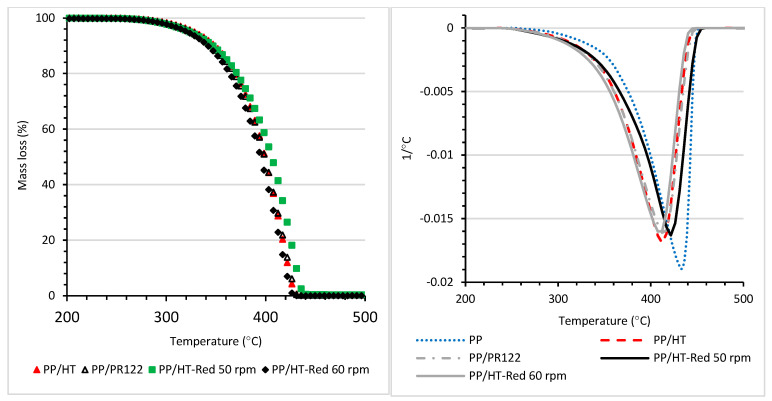
TGA and DTGA plots of masterbatches containing PR122 pigment and modified HT-Red fillers.

The influence of the colored fillers on the thermal properties of the masterbatches was analyzed using the TGA method (Figure 10 and Figure 11).

It can be seen from the TGA and DTGA plots (Figure 10) that the incorporation of both HT-Green 50 rpm and HT-Green 60 rpm instead of neat pigment PG7 improved the thermal stability of the masterbatch. This effect was stronger for the HT-Green filler prepared by using a higher speed of milling during the modification of layered double hydroxide. A reduced tendency of agglomeration for the HT-Green filler at 60 rpm and better dispersion in the masterbatch were responsible for the enhancement in the thermal stability of the masterbatch. Similarly, the incorporation of the colored HT-Red filler instead of the PR122 pigment improved the thermal stability of the masterbatch (Figure 11). Here, the shift in the temperature at which the maximum weight loss occurred toward higher values of temperature was stronger after the incorporation of the HT-Red filler at 50 rpm. The temperatures at which 2, 5, 10, 20% (T_2%_, T_5%_, T_10%_, and T_20%_) and the maximum weight loss, T_max_, occurred are compiled in Table 7.

The temperature at which the maximum weight loss, T_max_, of the masterbatches occurred was lower compared with that of PP pallets used during the preparation of the masterbatches. This resulted from the previously described thermo-mechanical degradation of the polypropylene base during the extrusion and the preparation of the masterbatch. The incorporation of the modified, colored filler HT-Green instead of neat pigment PG7 strongly shifted the temperatures of 2, 5, 10, and 20% of weight loss toward higher values of temperature. The shift in the temperatures of 5% of weight loss, T_5%_, for the masterbatch after the replacement of neat PG7 pigment with the HT-Green filler at 50 rpm was 5 °C and after the incorporation of the HT-Green filler at 60 rpm it was 19 °C. The enhancement of thermal stability after incorporation into polymeric material layered double hydroxide was reported by other authors [28], but here, for the masterbatch containing HT-Green at 60 rpm it also resulted from the synergistic effect of the filler and pigment. The higher thermal stability of the masterbatch containing HT-Green is a great processing advantage. The incorporation of HT-Red instead of the neat PR122 pigment, to a lesser extent, influenced the temperatures of 2 and 5% of the weight loss, T_2%_ and T_5%_. The incorporation of the HT-Red filler at 50 rpm instead of the neat PR122 pigment shifted the temperatures of 10 and 20% of the weight loss. The modified HT-Red hybrid colored filler at 50 rpm had the lowest polarity index, γ^P^/γ, as determined by us via the measurement of surface free energy. The enhancement of the non-polar character and the decrease in the polar composition of HT-Red 50 rpm with respect to unmodified HT and PR122 was, here, important from the point of view of mixing them with non-polar polypropylene, PP. This resulted in better homogenization with polypropylene during mixing and influenced the thermal properties of the obtained PP/HT-Red 50 rpm masterbatch causing the strongest changes in T_max_ °C. Thus, the temperature at the maximum weight loss of the masterbatch occurred after the replacement of the neat pigment PR122 by HT-Red 50 rpm shifted toward higher values of temperature of about 8 °C.

The influence of the modified colored fillers on the melting, T_m_, and crystallization, T_c_, temperatures was analyzed. The melting temperatures, T_m_, and the calculated degree of crystallinity for the masterbatches produced via extrusion and cooled using air are compiled in Table 8. Additionally, the melting temperature, T_m_, of the polypropylene processed in a similar way was added. The melting temperatures of all produced masterbatches did not vary significantly. Further, the difference in melting of the produced masterbatches and polypropylene was not observed. This is an advantage when considering the application of the masterbatches as the coloring system for the polypropylene products. The influence of the additives on the degree of the crystallinity, χ_c_, was observed. The addition of the neat pigments PG7 and PR122 enhanced the degree of crystallinity, χ_c_, of the masterbatch compared with that of the neat polypropylene or material containing layered double hydroxide. Similarly, the degree of crystallinity, χ_c_, increased after the addition of the colored fillers compared with that of the neat polypropylene or material containing unmodified layered double hydroxide. Here, it should be noted that the produced masterbatches were cooled using air, and thus the calculated values of degree of crystallinity, χ_c_, showed the percentage of the crystalline phase after the process of masterbatch production.

To estimate the influence of the additives on the crystallization temperature, T_c_, and melting temperature, T_m_, the samples were additionally analyzed after removing the thermal history of samples and after crystallization at a defined speed of cooling. In Table 9, the crystallization, T_c_, and melting, T_m_, temperatures determined via DSC are compiled, together with the calculated values of the degree of crystallinity, χ_c_, for samples cooled and further heated at a speed of 10 °C·min^−1^. All DSC plots for the studied materials are compiled in the Supporting Information (Figures S10–S16).

**Figure 12:**
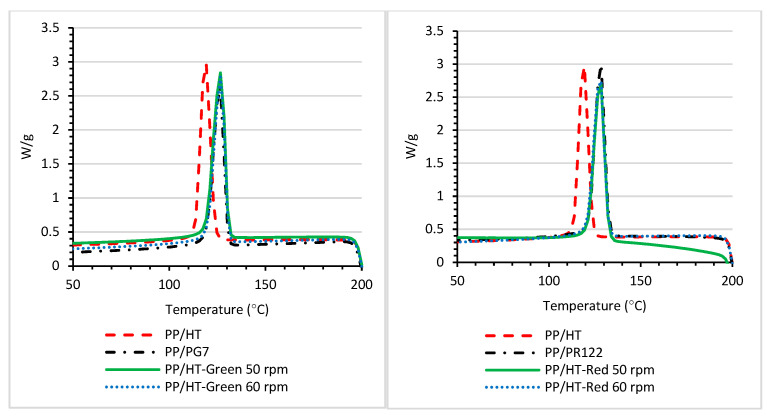
DSC plots of the cooling step for masterbatches containing the PG7 and PR122 pigments and modified fillers HT-Green and HT-Red. A speed of cooling of 10 °C·min^−1^ was used.

Figure 12 shows the DSC cooling step for the studied materials. The addition of both pigments, PG7 and PR122, or the colored modified HT-Green and HT-Red fillers strongly influenced the crystallization temperature, T_c_, of the masterbatches compared with that of the sample containing unmodified layered double hydroxide. Here, higher temperatures of crystallization, T_c_, were detected. The shift in crystallization temperatures between samples PP/HT and PP/PG7 was about 7.5 °C, and between samples PP/HT and PP/PR122 it was about 9.9 °C. Similarly, the addition of the colored modified HT-Green and HT-Red fillers shifted the crystallization temperatures towards higher value of temperature. The impact of the color additives on the melting temperatures, T_m_, was not so evident. It is well known that cooling speed is a factor influencing the formed crystalline phase [9]. The determined the degree of crystallinity, χ_c_, after the removal of the thermal history of the sample was higher for the sample containing unmodified layered double hydroxide. Both fillers and pigments could act as nucleating agents of polypropylene enhancing the formation of the crystalline phase [7,8]. The presence of the layered double hydroxide was the main factor influencing the crystallization of the masterbatch. However, more in-depth studies are needed to determine the impact of the surface covering of the layered double hydroxide HT by chosen pigments on the crystallization of the masterbatches.

### 3.4. The Influence of Hybrid Pigment on the Color Profile of Masterbatch

**Figure 13:**
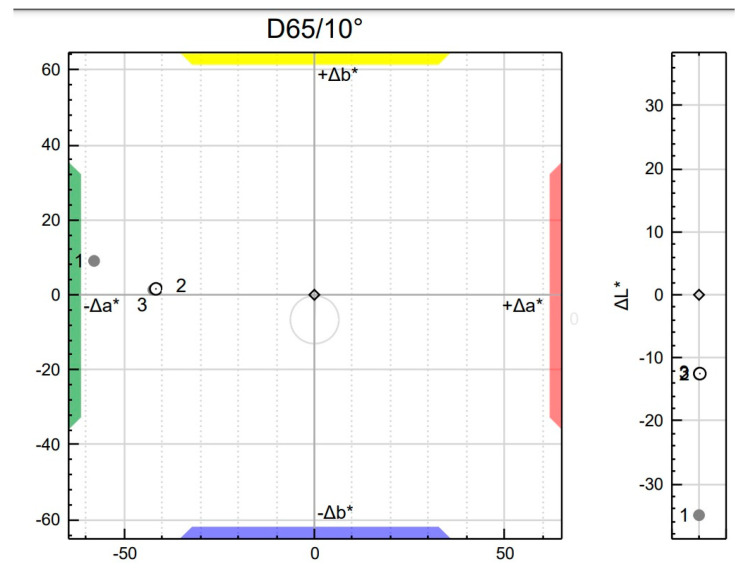
Color coordinates for modified PG7.

Figure 13 shows 1-neat PG7, 2-HT-Green 50 rpm and 3-HT-Green 60 rpm, respectively. Based on the coordinates, a significant change in the color of the modified pigments in relation to the hydrotalcite can be seen, which was obvious and visible to the naked eye. Comparing the color change of HT-Green to that of the unmodified pigment, an increase in brightness could be seen via the increasing L parameter and shifting X coordinate toward a red color.

**Figure 14:**
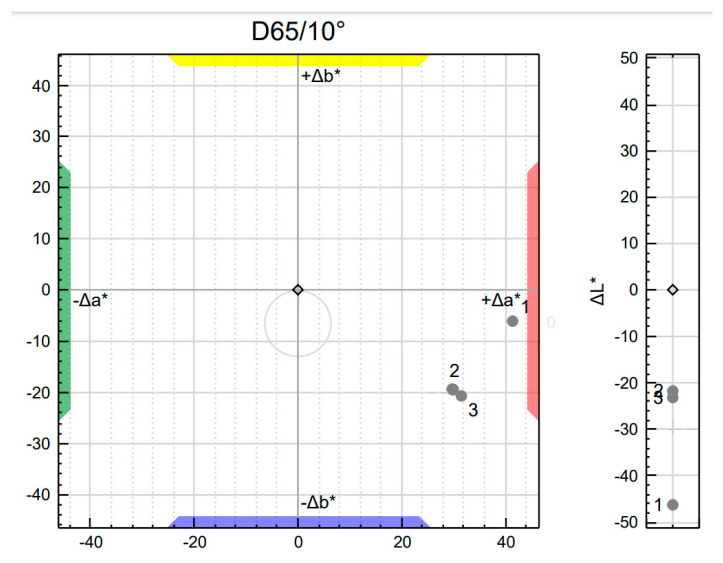
Color coordinates for modified PR122.

Figure 14 shows 1-neat PR122, 2-HT-Red 50 rpm and 3-HT-Red 60 rpm. Comparing the color change in HT-Red to that of the unmodified pigment, an increase in brightness could be seen via increasing the L parameter and shifting X coordinate toward a green color and that of the a coordinate toward a bluer color.

## 4. Conclusions

The surface of the layered double hydroxide was modified using quinacridone and phthalocyanine green pigments using the optimized milling process. The applied method influenced the size of the aggregates formed by layered double hydroxide HT.

The tensiometer measurement showed a decrease in the polar component of surface free energy, γ^P^, after the modification of the hydrotalcite with both pigments when a 50 rpm speed of milling was used. This is advantageous from the point of view of mixing modified LDH with non-polar polypropylene.

It is worth noting that hydrotalcite modified using phthalocyanine green, HT-Green, are characterized by higher thermal stability compared with that of pure hydrotalcite, which has been proven using the thermogravimetric technique. After analyzing the results obtained via the DTGA measurement, the temperature at which 5, 10 and 20% mass loss occurred was determined to be shifted to a higher value of temperature. It turns out that the PG7 pigment has a higher thermal resistance than PR122 does, and therefore, the modified HT-Green color filler also has a higher thermal resistance compared to that of HT-Red.

The modification of hydrotalcite with pigments slightly influenced the rheological properties of the masterbatches. It was noticed that this additive slightly increased the rheological parameters such as the storage shear modulus, G’, and loss shear modulus, G″. This is very beneficial from an industrial point of view and from that of the future application of a new modified colored filler instead of the previously used pure pigments in masterbatch formulation. Further analyzing the rheological properties of the tested systems, it can be seen that the storage modulus, loss modulus and loss factor tan, *δ*, value of the PP/PR122 system have higher values than those in the case of the PP/PG7 system. Taking into account the system containing hydrotalcite modified with phthalocyanine green, it was noticed that PP/HT-Green had a stronger influence on the values of the storage modulus, loss modulus and loss factor tan, *δ*, where HT–pigment mixtures were prepared for 15 min and at a mixing speed of 50 rpm.

The addition of HT–pigments facilitated the relaxation of melted PP/HT–pigment system compared with that of pure polypropylene or PP/HT composites. Hydrotalcite modified by using phthalocyanine green, HT-Green 60 rpm, had the strongest effect on the relaxation of the melted masterbatch. The lower values of characteristic relaxation times calculated based on the viscosity Cole–Cole plots were reported. Similarly, also, other models applied to calculate the relaxation times (the Maxwell model and continuous relaxation model) showed a shortening of the relaxation times.

The positive effect of modification on the thermal stability of the polypropylene masterbatches was noticed, and higher temperatures of the maximum weight loss, T_max_, were determined for PP/HT–pigment masterbatches compared to those of masterbatches with PP/HT. The maximum weight loss temperature, T_max_, of the masterbatch after the replacement of the red quinacridone pigment PR122 via modified HT-Red 50 rpm shifted toward higher values of temperature of about 8 °C.

## Figures and Tables

**Table 1:** Range of the aggregate size formed in the polar water medium for the modified, colored fillers.

Sample	Range of Aggregates Size (nm) *	Size of the Main Fraction (nm) *	Percentage as Number of Main Fraction (%) *
Unmodified HT	940–2118	1332	38.0
HT-Green 50 rpm	1484–7456	3091	27.0
HT-Green 60 rpm	1718–7456	4145	26.7
HT-Red 50 rpm	1246–1589	1471	24.1
HT-Red 60 rpm	1351–1893	1763	23.4

* Measurements were taken in the polar water medium.

**Table 2:** Range of the aggregate size formed in the non-polar paraffin oil medium for the modified, colored fillers.

Sample	Range of Aggregates Size (nm) *	Size of the Main Fraction (nm) *	Percentage by Number of Main Fraction (%) *
Unmodified HT	995–1545	1270	35.6
HT-Green 50 rpm	1152–2780	2376	27.8
HT-Green 60 rpm	2073–2780	2586	54.9
HT-Red 50 rpm	1152–1790	1438	34.3
HT-Red 60 rpm	1152–2073	1561	33.1

* Measurements were taken in the non-polar paraffin oil medium.

**Table 3:** Calculated values of surface free energy, *γ*, dispersive, *γ*^d^, and polar, *γ*^p^, components of surface free energy and the polarity index, *γ*^p^/*γ*.

Sample	*γ*^p^ dynes·cm^−1^	*γ*^d^ dynes·cm^−1^	*γ* dynes·cm^−1^	*γ*^p^/*γ*
Unmodified HT	3.0	16.1	19.2	0.16
PG7	5.8	17.2	23.0	0.25
HT-Green 50 rpm	2.7	21.8	24.5	0.11
HT-Green 60 rpm	8.1	10.5	18.6	0.44
PR122	1.0	11.9	12.8	0.08
HT-Red 50 rpm	0.5	10.2	10.7	0.05
HT-Red 60 rpm	8.9	18.9	27.8	0.32

**Table 4:** The temperatures at 5, 10 and 20% of weight loss, T_5%_, T_10%_, and T_20%_, and the temperatures of the maximum of the DTGA peaks, T_max_.

	T_5%_ (°C)	T_10%_ (°C)	T_20%_ (°C)	T_max_ (°C)
PG7	598	622	649	697
PR122	532	559	583	601; 733
HT	211	238	337	241; 430
HT−Green 50 rpm	217	244	355	241; 431; 625
HT−Green 60 rpm	214	241	352	241; 430; 622
HT−Red 50 rpm	181	223	355	226; 430; 622
HT−Red 60 rpm	181	223	352	229; 433; 625

**Table 5:** The viscoelastic parameters of masterbatches at 200 °C.

Composition	G′_LVR_ (Pa)	G″_LVR_ (Pa)	Tan δ (−)
PP	4614 ± 49	8371 ± 57	1.81 ± 0.01
PP/HT	3292 ± 28	6056 ± 44	1.84 ± 0.01
PP/PG7	3476 ± 85	6478 ± 101	1.86 ± 0.02
PP/HT-Green 50 rpm	4360 ± 55	8125 ± 93	1.86 ± 0.01
PP/HT-Green 60 rpm	4154 ± 59	7724 ± 113	1.86 ± 0.04
PP/PR122	3665 ± 115	7207 ± 159	1.97 ± 0.02
PP/HT-Red 50 rpm	5413 ± 95	9264 ± 84	1.71 ± 0.02
PP/HT-Red 60 rpm	3895 ± 88	6938 ± 92	1.78 ± 0.03

G′—storage shear modulus; G″—loss shear modulus; δ—loss factor tan δ measured at 200 °C at an angular frequency of 10 rad·s^−1^.

**Table 6:** The characteristic relaxation time, τ_m_ (s), and the zero-shear viscosity, η_0_ (Pas).

Composition	τ_m_ (s)	η_0_ (Pas)
PP	1.123	3041
PP/HT	0.892	2120
PP/PG7	1.001	1728
PP/HT-Green 50 rpm	0.708	2225
PP/HT-Green 60 rpm	0.795	1789
PP/PR122	1.013	1829
PP/HT-Red 50 rpm	0.885	1942
PP/HT-Red 60 rpm	0.889	2014

**Table 7:** The temperatures at which 2, 5, 10 and 20% of weight loss, T_2%_, T_5%_, T_10%_, and T_20%_, occurred and the temperatures of the maximum of the DTGA peaks, T_max_.

	T_2%_ °C	T_5%_ °C	T_10%_ °C	T_20%_ °C	T_max_ °C
PP	316	337	361	385	433
PP/HT	305	328	347	370	427
PP/PG7	291	319	342	361	428
PP/HT-Green 50 rpm	296	324	347	370	431
PP/HT-Green 60 rpm	310	338	356	379	431
PP/PR122	300	323	342	366	429
PP/HT-Red 50 rpm	300	324	347	370	437
PP/HT-Red 60 rpm	300	324	342	366	427

**Table 8:** The melting temperatures, T_m_, the values of the enthalpy of melting, ΔH_m_, and the degree of crystallinity, χ_c_, of the produced masterbatches.

	T_m_ (°C) *	ΔH_m_ (J·g^−1^) *	χ_c_ (%) *
PP	170	86.70	41.9
PP/HT	169	84.58	40.9
PP/PG7	169	92.86	44.9
PP/HT-Green 50 rpm	168	100.79	48.8
PP/HT-Green 60 rpm	168	95.32	46.1
PP/PR122	167	101.68	49.2
PP/HT-Red 50 rpm	169	94.16	45.6
PP/HT-Red 60 rpm	168	102.92	49.8

* The parameters were determined via DSC for the produced masterbatches.

**Table 9:** The crystallization, T_c_, and melting, T_m_, temperatures, the values of crystallization, ΔH_c_, and the melting ΔH_m_ enthalpy, and the degree of crystallinity, χ_c_.

	T_c_ (°C) *	H_c_ (J·g^−1^) *	T_m_ (°C) *	ΔHm (J·g^−1^) *	χ_c_ (%) *
PP/HT					
PP/PG7	119.7	96.47	166.5	91.95	44.5
PP/HT-Green 50 rpm	127.2	92.95	170.8	81.7	39.6
PP/HT-Green 60 rpm	128.0	102.14	169.1	89.24	43.2
PP/PR122	127.8	95.24	170.5	84.26	40.8
PP/HT-Red 50 rpm	129.6	100.94	170.3	87.74	42.5
PP/HT-Red 60 rpm	129.1	98.95	172.2	83.45	40.4
	129.1	98.63	169.7	88.99	43.1

* The parameters were determined via DSC for the produced masterbatches after removing, during the measurement, the thermal history of the sample. An applied speed of cooling and heating of 10 °C·min^−1^ was used.

## Data Availability

Data available on request.

## Supplementary Materials

The following supporting information can be downloaded at https://www.mdpi.com/article/10.3390/ma16186243/s1. Figure S1: XRD diffraction patterns of layered double hydroxide Pural MG70; Figure S2: The optical microscope images of hydrotalcite, HT, pigment Green 7, phthalocyanine green, PG7 (c), and pigment Red 122, 2,9-dimethylquinacridone,((2,9-dimethyl-5,12-dihydroquinolino[2,3-b]acridine-7,14-dione), PR122 (d), at a magnification of 1000×.; Figure S3: The optical microscope images of hydrotalcite, HT, modified with pigment Green 7, phthalocyanine green, PG7, after milling at various speeds: 50 rpm (a,b); 60 rpm (c,d). The images were taken at a magnification of 500× (a–c) and 1000× (b–d); Figure S4: The optical microscope images of hydrotalcite, HT, modified with pigment Red 122, 2,9-dimethylquinacridone,((2,9-dimethyl-5,12-dihydroquinolino[2,3-b]acridine-7,14-dione), PR122, after milling at various speeds: 50 rpm (a,b); 60 rpm (c,d). The images were taken at a magnification of 500× (a) and 1000x (b–d); Figure S5: The DLS plots of aggregate sizes as a function of percentage by volume for hydrotalcite and modified HT-Green fillers; Figure S6: The DLS plots of aggregate sizes as a function of percentage by volume for hydrotalcite and modified HT-Red fillers; Figure S7: The viscoelastic properties, the storage shear modulus, G′ (Pa), and loss shear modulus, G″ (Pa), for PP masterbatches based on the PG7 pigment and HT-Green; Figure S8: The viscoelastic properties, storage shear modulus, G′ (Pa), and loss shear modulus, G″ (Pa), for PP masterbatches based on the PR122 pigment and HT-Red; Figure S9: The values of complex viscosity as a function of frequency at 220 °C for the palettes of polypropylene and the masterbatches extruded and processed under similar conditions as those of the polypropylene masterbatches; Table S1: Values of relaxation modulus, G_i_ (Pa), and relaxation times, λ_i_ (s), calculated using Maxwell models; Table S2: Values of relaxation modulus, G_i_ (Pa), and relaxation times, λ_i_ (s), calculated using Maxwell models; Figure S10: DSC plots for sample PP/HT; Figure S11: DSC plots for sample PP/PG7; Figure S12: DSC plots for sample PP/PR122; Figure S13: DSC plots for sample PP/HT-Green 50 rpm; Figure S14: DSC plots for sample PP/HT-Green 60 rpm; Figure S15: DSC plots for sample PP/HT-Red 50 rpm; Figure S16: DSC plots for sample PP/HT-Red 60 rpm.
