# Supplementary material for: Polypropylene Color Masterbatches Containing Layered Double Hydroxide Modified with Quinacridone and Phthalocyanine Pigments—Rheological, Thermal and Application Properties
Source: Materials (Basel). 2023 Sep 16;16(18):6243. doi: 10.3390/ma16186243 (PMC10532881; doi:10.3390/ma16186243)
Supplement: Supplementary file 1 [file materials-16-06243-s001.zip › materials-2610009-supplementary.pdf]

Supporting Information

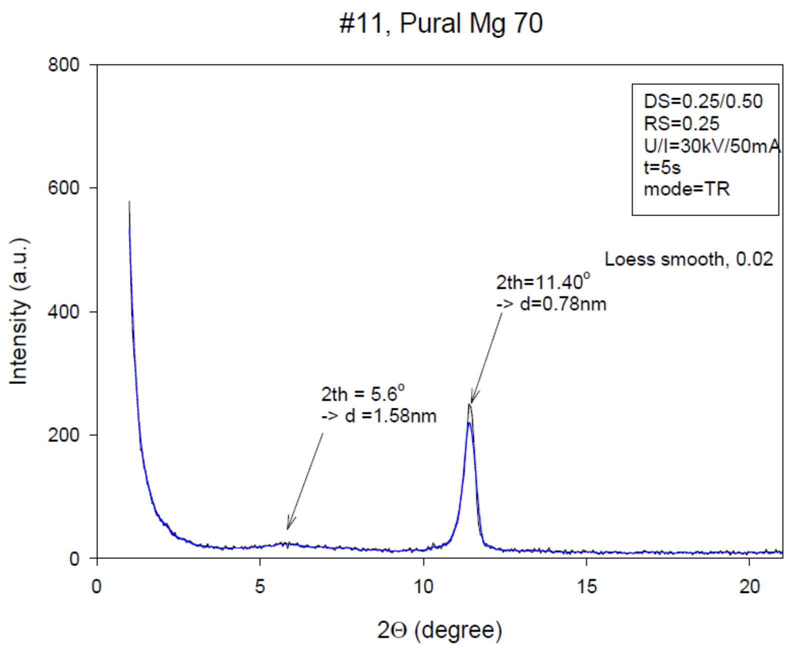

Figure S1. XRD diffractions patterns of layered double hydroxide Pural MG70.

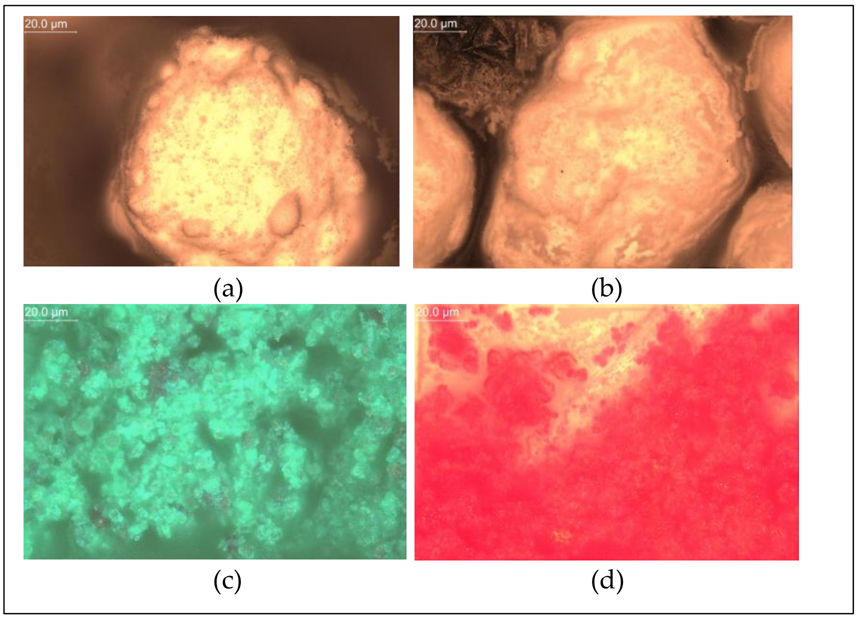

Figure S2. The optical microscope images of hydrotalcite HT (a-b), pigment Green 7, phthalocyanine green, PG7 (c) and pigment Red 122, 2,9-dimethylquinacridone,((2,9-dimethyl-5,12-dihydroquinolino[2,3-b]acridine-7,14-dione), PR122 (d) at magnification 1000x. The optical microscope images were done by using the Opta-Tech Lab40 microscope (Opta-Tech, Warsaw, Poland) connected with digital camera Mi6 with 6 megapixel sensor-IMX178, Resolution -3072 x 2048 (Opta-Tech, Warsaw, Poland) and computer program Capture 2.3 (Opta-Tech, Warsaw, Poland).

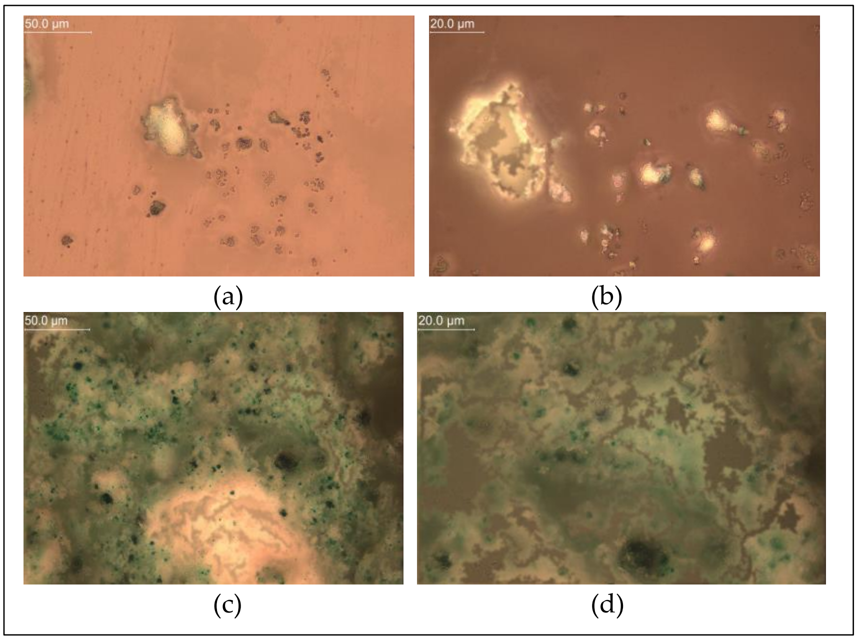

Figure S3. The optical microscope images of hydrotalcite HT modified by pigment Green 7, phthalocyanine green, PG7 after milling at various speed 50 rpm (a-b); 60 rpm (c-d). The images were done at magnification 500x (a-c) and 1000x (b-d). The optical microscope images were done by using the Opta-Tech Lab40 microscope (Opta-Tech, Warsaw, Poland) connected with digital camera Mi6 with 6 megapixel sensor-IMX178, Resolution -3072 x 2048 (Opta-Tech, Warsaw, Poland) and computer program Capture 2.3 (Opta-Tech, Warsaw, Poland).

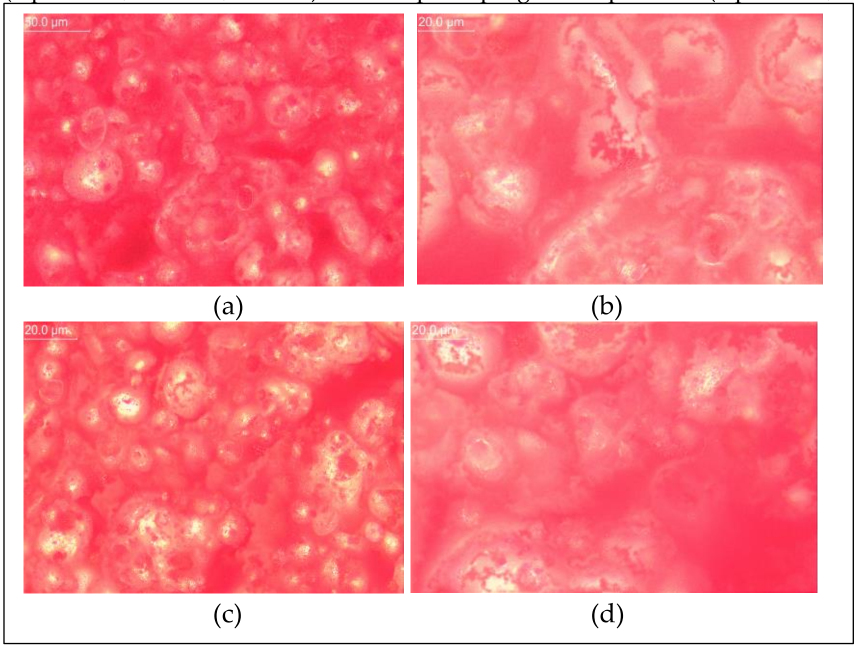

Figure S4. The optical microscope images of hydrotalcite HT modified by pigment Red 122, 2,9-dimethylquinacridone,((2,9-dimethyl-5,12-dihydroquinolino[2,3-b]acridine-7,14-dione), PR122 after milling at various speed 50 rpm (a-b); 60 rpm (c-d). The images were done at magnification 500x (a) and 1000x (b-d). The optical microscope images were done by using the Opta-Tech Lab40 microscope (Opta-Tech, Warsaw, Poland) connected with digital camera Mi6 with 6 megapixel sensor-IMX178, Resolution -3072 x 2048 (Opta-Tech, Warsaw, Poland) and computer program Capture 2.3 (Opta-Tech, Warsaw, Poland).

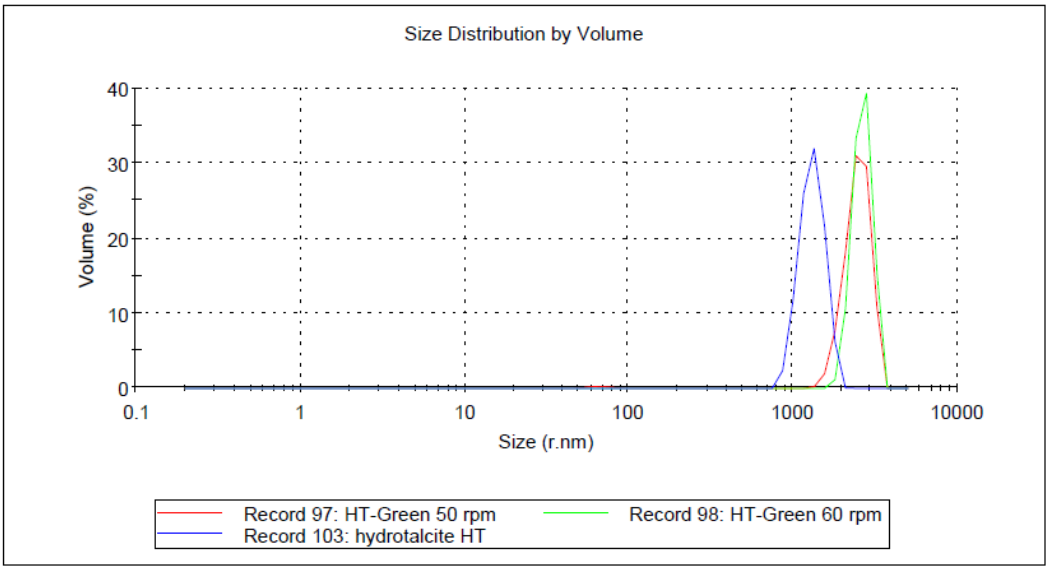

Figure S5. The DLS plots of aggregates sizes as a function of percentage by volume for hydrotalcite and modified HT-Green fillers.

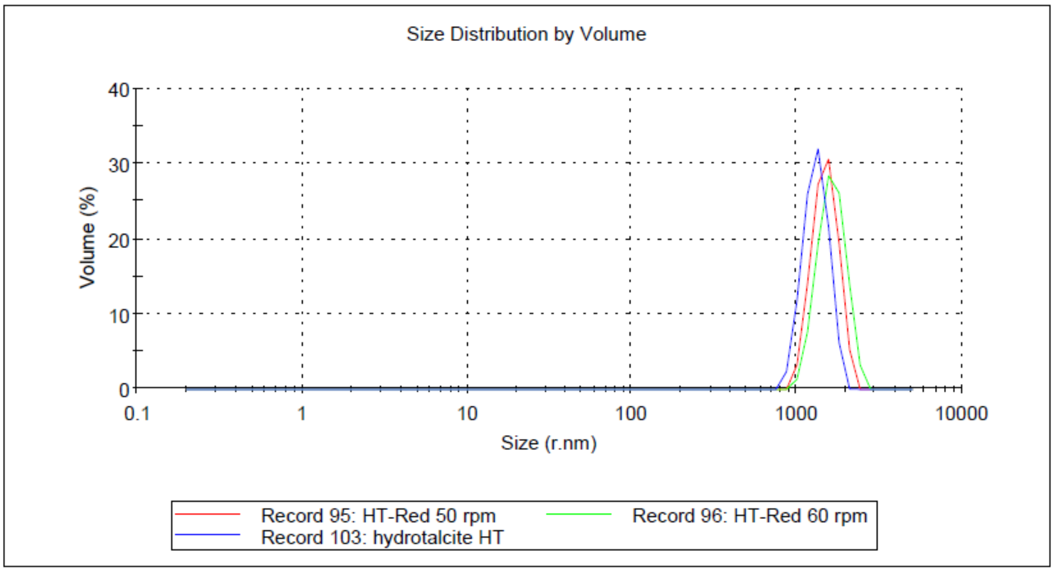

Figure S6. The DLS plots of aggregates sizes as a function of percentage by volume for hydrotalcite and modified HT-Red fillers.

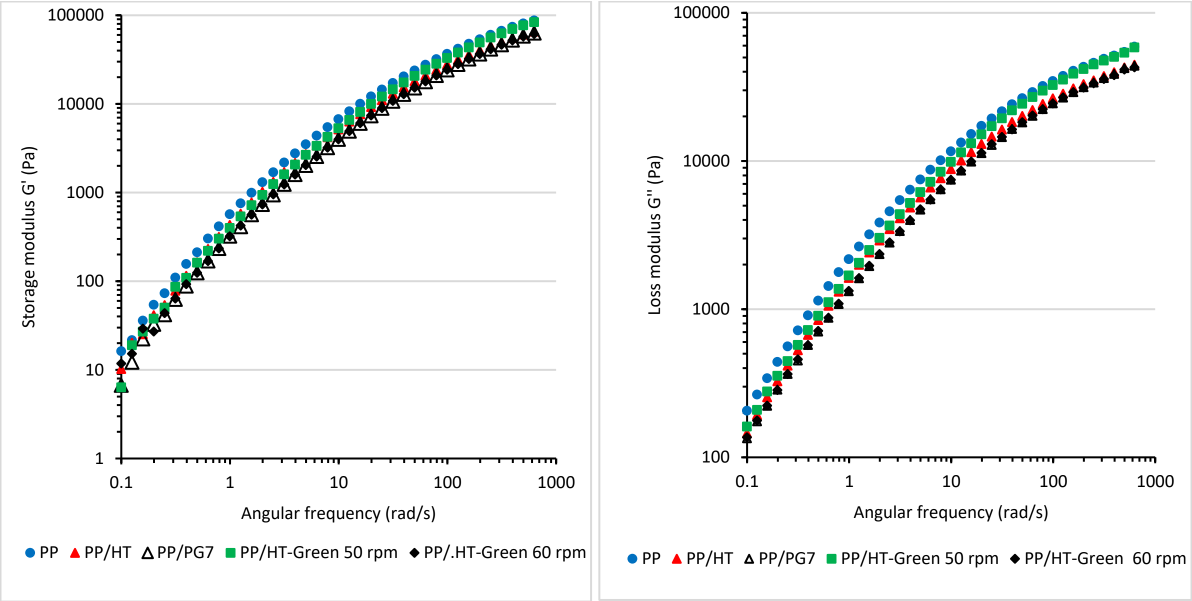

Figure S7. The viscoelastic properties, the storage shear modulus G′ (Pa), loss shear modulus G″ (Pa) for PP masterbatches based on the PG7 pigment and HT-Green.

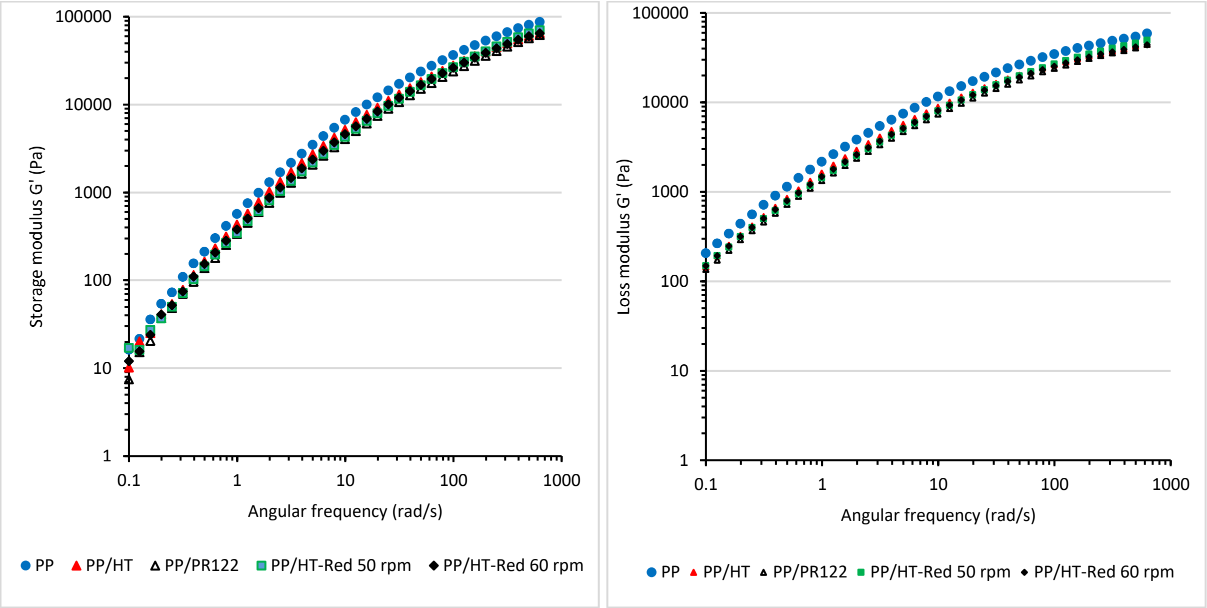

Figure S8. The viscoelastic properties, storage shear modulus G′ (Pa), loss shear modulus G″ (Pa) for PP masterbatches based on the PR122 pigment and HT-Red.

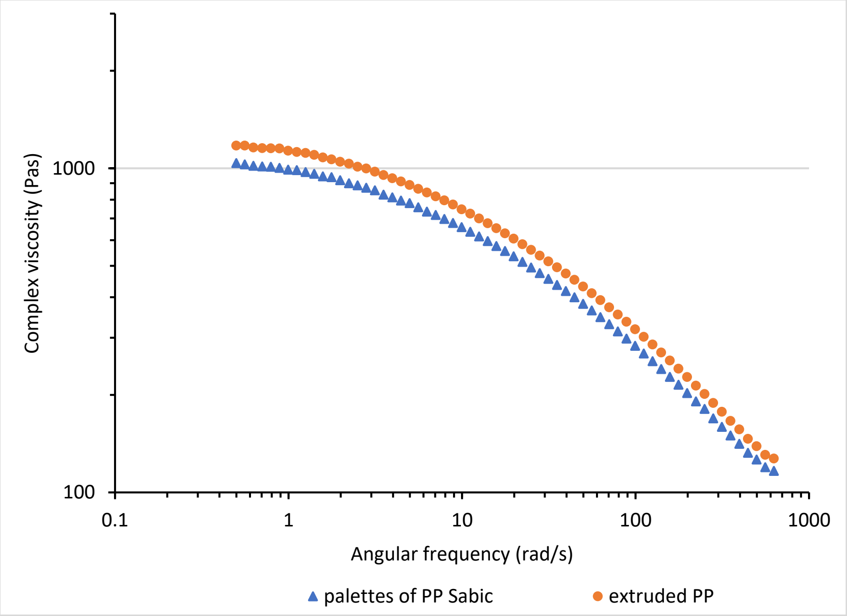

Figure S9. The values of the complex viscosity as a function of frequency at 220°C for the palettes of polypropylene and the extruded and processed under similar conditions as masterbatches polypropylene.

Table S1. Values of relaxation modulus G_i (Pa) and relaxation times λ_i (s) calculated using Maxwell models.

	PP		PP+HT		PP+6010		PP+mod.HT/6010 50 rpm	
n	Relaxation time λ (s)	Relaxation modulus G_i (Pa)	Relaxation time λ (s)	Relaxation modulus G_i (Pa)	Relaxation time λ (s)	Relaxation modulus G_i (Pa)	Relaxation time λ (s)	Relaxation modulus G_i (Pa)
1	$7.59\cdot10^{-4}$	117742	$7.09\cdot10^{-4}$	95257	$1.03\cdot10^{-3}$	80623.3	$1.01\cdot10^{-3}$	101833
2	$5.36\cdot10^{-3}$	42435.1	$5.20\cdot10^{-3}$	32029.6	$6.43\cdot10^{-3}$	27556.7	$5.96\cdot10^{-3}$	36253.1
3	0.023337	19750.4	0.022822	15288.3	0.028635	11716.8	0.023879	16256
4	0.093555	7096.12	0.095852	5393.16	0.128618	3454.32	0.089001	5472
5	0.389827	1831.1	0.411365	1341.33	0.5859	666.816	0.356607	1462.97
6	1.7081	285.786	1.75722	190.623	2.80499	74.9226	1.87767	207.584
R^2	0.9997		0.9995		0.9994		0.9999	

Table S2. Values of relaxation modulus G_i (Pa) and relaxation times λ_i (s) calculated using Maxwell models.

	PP+mod.HT/6010 60 rpm		PP+48010		PP+mod.HT/48010 50 rpm		PP+mod.HT/48010 60 rpm	
n	Relaxation time λ (s)	Relaxation modulus G_i (Pa)	Relaxation time λ (s)	Relaxation modulus G_i (Pa)	Relaxation time λ (s)	Relaxation modulus G_i (Pa)	Relaxation time λ (s)	Relaxation modulus G_i (Pa)
1	$9.33\cdot10^{-4}$	80681.1	$8.97\cdot10^{-4}$	79190.7	1.10E-03	84423.2	80990.7	9.28E-04
2	$6.09\cdot10^{-3}$	29139.4	$5.37\cdot10^{-3}$	29083.1	6.10E-03	28573.7	28914.1	5.57E-03
3	0.027762	12117	0.021368	13448.2	0.023988	13071.3	13783.4	0.023052
4	0.122737	3505.86	0.080734	4658.98	0.09398	4417.98	4877.07	0.091561
5	0.535942	734.45	0.334557	1226.64	0.378628	1171.97	1300.49	0.377464
6	2.61887	90.5021	1.81506	161.362	1.94851	185.487	192.045	1.86439
R^2	0.9997		0.9992		0.9991		0.9994	

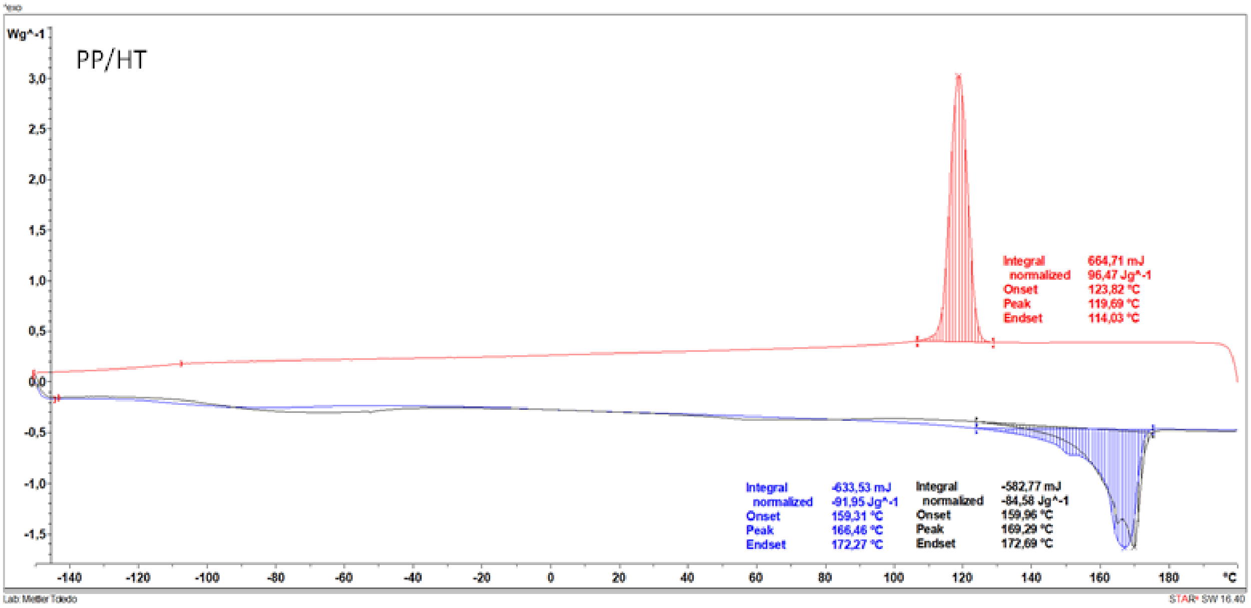

Figure S10. DSC plots for sample PP/HT.

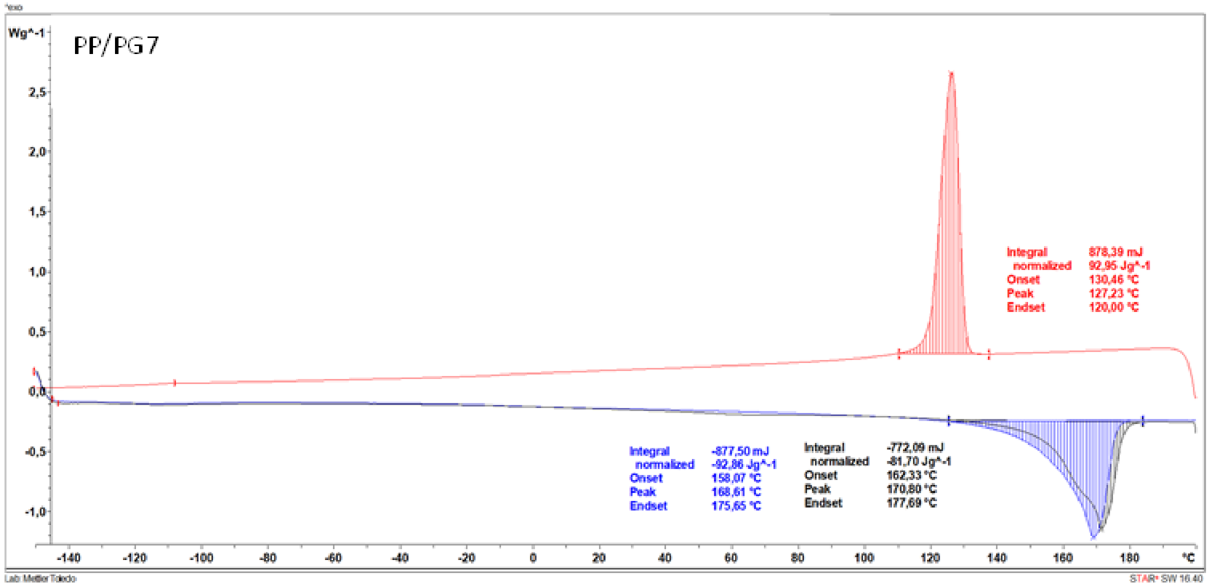

Figure S11. DSC plots for sample PP/PG7.

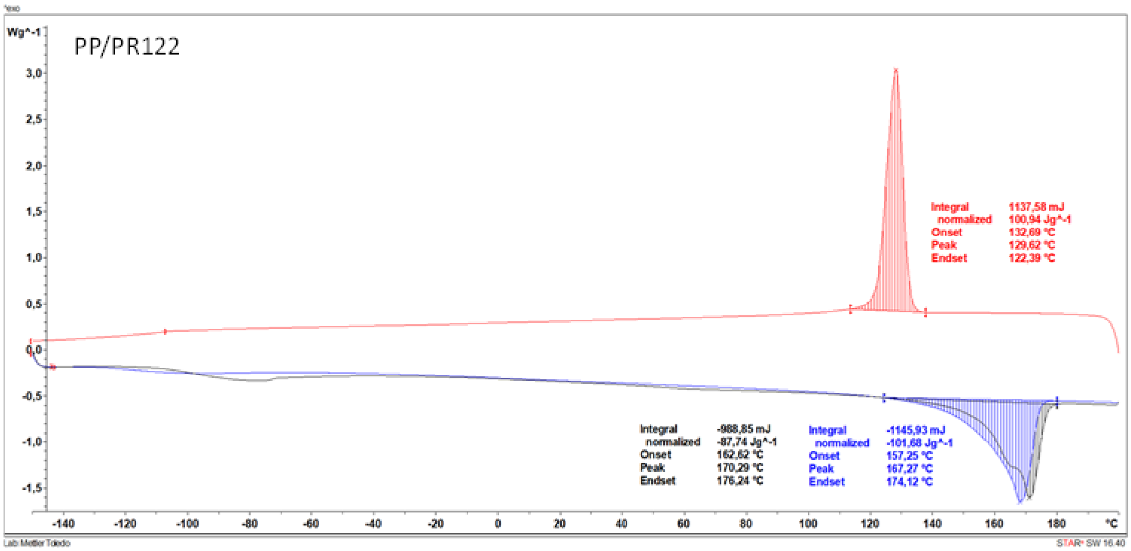

Figure S12. DSC plots for sample PP/PR122.

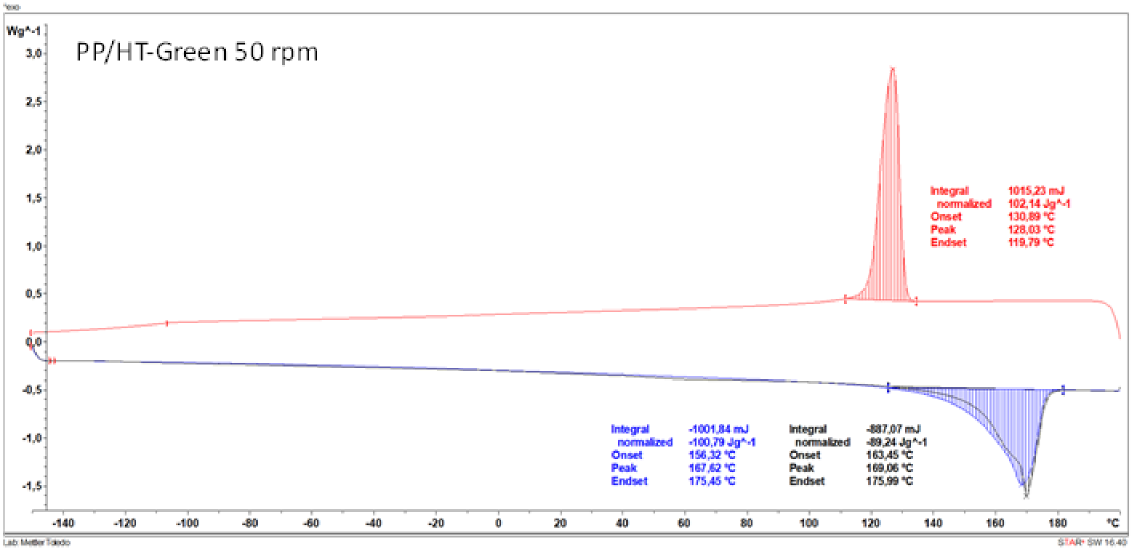

Figure S13. DSC plots for sample PP/HT-Green 50 rpm.

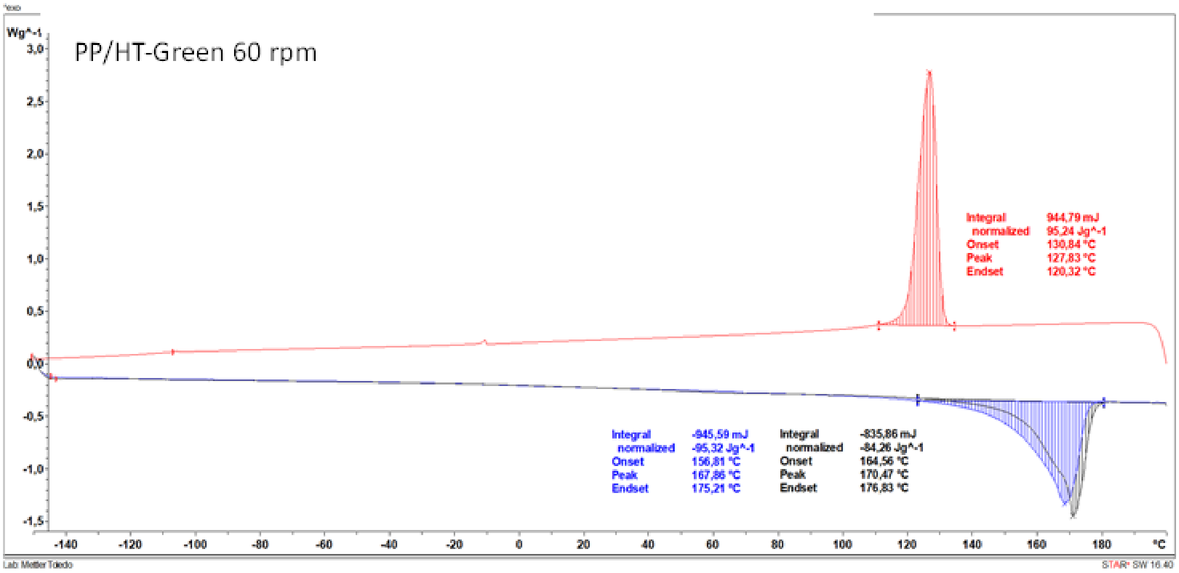

Figure S14. DSC plots for sample PP/HT-Green 60 rpm.

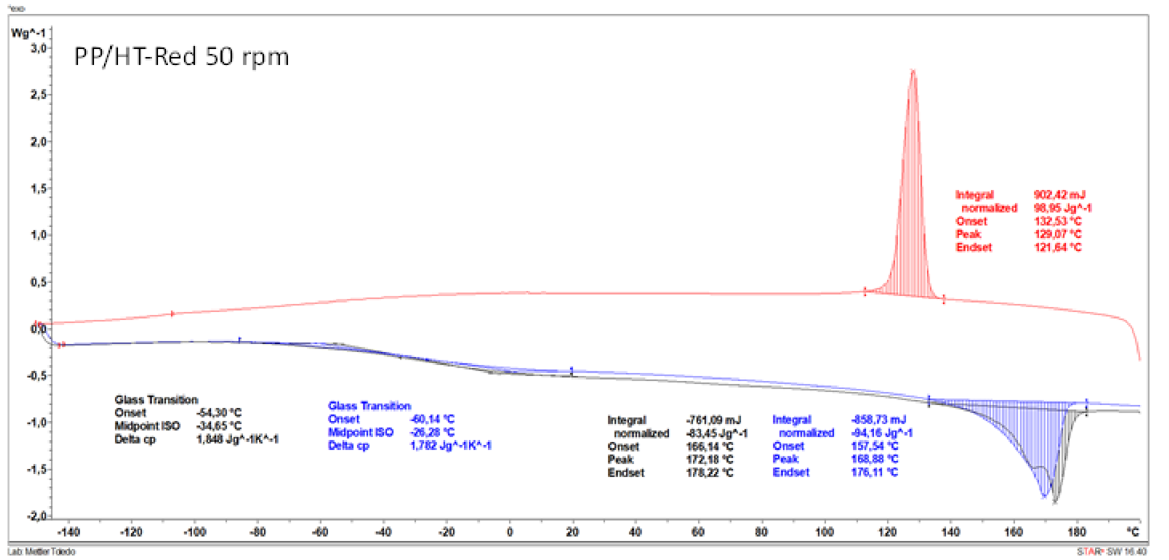

Figure S15. DSC plots for sample PP/HT-Red 50 rpm.

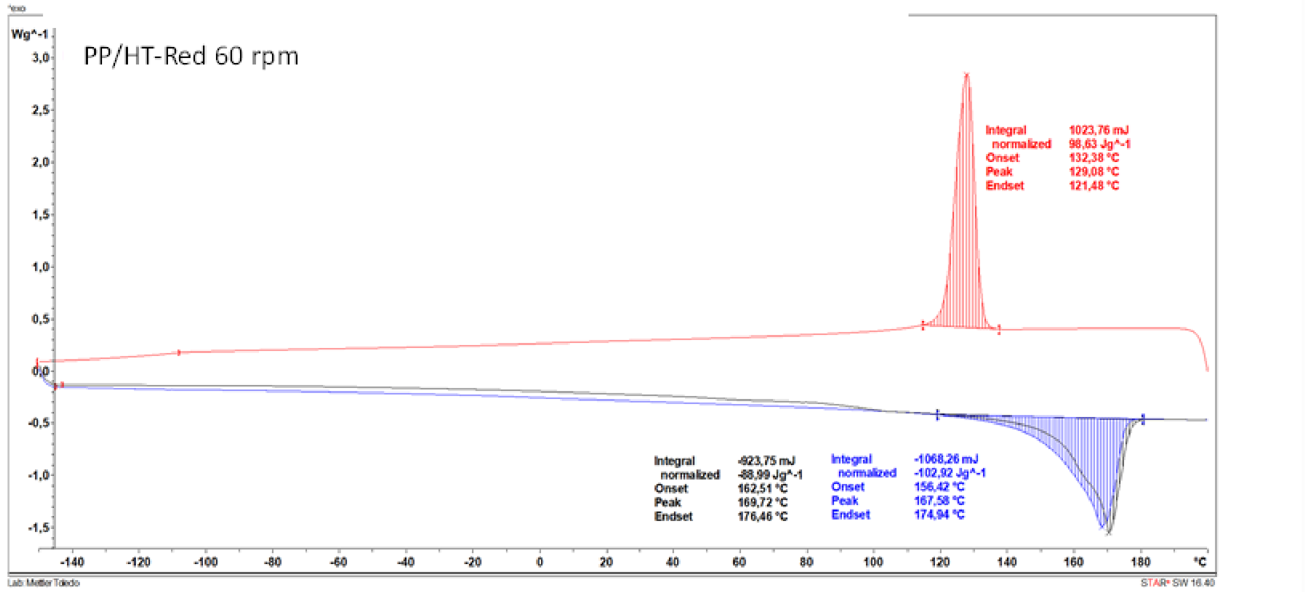

Figure S16. DSC plots for sample PP/HT-Red 60 rpm.
